# Glycoconjugations of Biomolecules by Chemical Methods

**DOI:** 10.3389/fchem.2020.570185

**Published:** 2020-11-04

**Authors:** Biswajit Sarkar, Narayanaswamy Jayaraman

**Affiliations:** Department of Organic Chemistry, Indian Institute of Science, Bangalore, India

**Keywords:** biomolecules, carbohydrates, glycoconjugations, lipids, nucleic acids, peptides, proteins, conjugations

## Abstract

Bioconjugations under benign aqueous conditions have the most promise to covalently link carbohydrates onto chosen molecular and macromolecular scaffolds. Chemical methodologies relying on C-C and C-heteroatom bond formations are the methods of choice, coupled with the reaction conditions being under aqueous milieu. A number of methods, including metal-mediated, as well as metal-free azide-alkyne cyclo-addition, photocatalyzed thiol-ene reaction, amidation, reductive amination, disulfide bond formation, conjugate addition, nucleophilic addition to vinyl sulfones and vinyl sulfoxides, native chemical ligation, Staudinger ligation, olefin metathesis, and Suzuki-Miyaura cross coupling reactions have been developed, in efforts to conduct glycoconjugation of chosen molecular and biomolecular structures. Within these, many methods require pre-functionalization of the scaffolds, whereas methods that do not require such pre-functionalization continue to be few and far between. The compilation covers synthetic methodology development for carbohydrate conjugation onto biomolecular and biomacromolecular scaffolds. The importance of such glycoconjugations on the functional properties is also covered.

## Introduction

The roles of carbohydrates in a multitude of biological functions are at the forefront of research in glycobiology (Varki et al., 2015). An outcome of decades of sustained efforts is a paradigm shift and distinct areas of studies, such as glycomics, emerged as a result. Synthetic glycoconjugates are sought in order to aid the uncovering of many biological functions of carbohydrates. Simultaneously, the application of synthetic glycoconjugates to overcome carbohydrate mediated aberrations or to benefit from carbohydrate mediated processes is a long-drawn area of research, an impressive area of development in this respect is the carbohydrate vaccines (Lang and Huang, 2020). The report of Goebel and Avery nearing a century ago emphasized the importance of conjugating sugars to a given protein, in order to confer antigenicity to the sugars (Goebel and Avery, 1929). Herein a chemical method was developed in which the sugar, in the form of *p*-nitrophenyl glycoside, was conjugated to the protein through diazotization reaction. Since then, glycoconjugation onto proteins have gone through many stages of developments and the conjugation chemistry has become one of the most intense areas of research. Yet another important development is the conjugation through Schiff base formation of sugar derived aldehyde with amines in proteins followed by a reduction, namely, the reductive amination (Gray, 1974). Given that the reducing end of oligo- and polysaccharides could be oxidized to the corresponding aldonic acid, amidation with proteins also became a resourceful method to conjugate sugars to proteins (Arakatsu et al., 1966). In order to improve upon the yields of the conjugations, spacers or linkers that connect the sugar component with the protein reactive sites became common (Lemieux et al., 1975).

Although many such methods were reported in a sustained manner, developing new conjugation chemistry was long-awaited until 2–3 decades ago. Conjugations that occur reagent-less, under physiological pH and photochemical stimuli, metal-mediated, and metal-free conditions became cornerstones in the developments. Expanding these frontiers is necessary in order to extend the methods that can occur in a completely cellular environment and also in *in vivo* conditions. Most such developments pertain to derivatizing the underlying protein, either as a scaffold or as an acceptor of the sugar to aid their functional modifications. Apart from glycoconjugation of proteins, biomolecules, namely lipids and nucleic acids, have also drawn interest in the field of conjugation chemistry.

Apart from classical methods based on amidation and reductive amination, many newly evolved conjugations pertaining to native chemical ligation, Staudinger ligation, azide-alkyne cycloaddition reactions, thiol-ene reaction, metathesis reactions, Suzuki-Miyaura reactions, vinyl sulfone reactions, and vinyl sulfoxide conjugations are discussed. Many contemporary methods might be considered as being stuck in the early stages, having come to the forefront of conjugation reactions in less than a couple of decades (Boutureira and Bernardes, 2015). Needless to mention, the conjugation chemistry has witnessed far more reports and examples than those fully covered in this article.

### Amidation

Primary amine in lysine side chain is a resourceful functionality for amidation reactions with carboxylic acid-containing sugars and their derivatives. An early known method of installing carboxylic acid in the sugar component is to oxidize the sugar lactol to the corresponding aldonic acid and utilize the amidation method through activation of acid with activating agents. The report of Lönngren, Goldstein, and Niederhuber showed aldonic acid coupling to bovine serum albumin (BSA) protein, mediated by *N*-(3-dimethylaminopropyl)-*N*′-ethylcarbodiimide (EDC) at acidic pH. Molar ratio of aldonic acid to protein was found to be important, as much as 9 molar equivalents of aldonic acid to each lysyl residue in the protein afforded amide bond formation with 45 of 59 available lysines in BSA. Mono-, di, and tri-saccharide aldonic acids were used in the study (Lönngren et al., 1976).

**Scheme 1:**
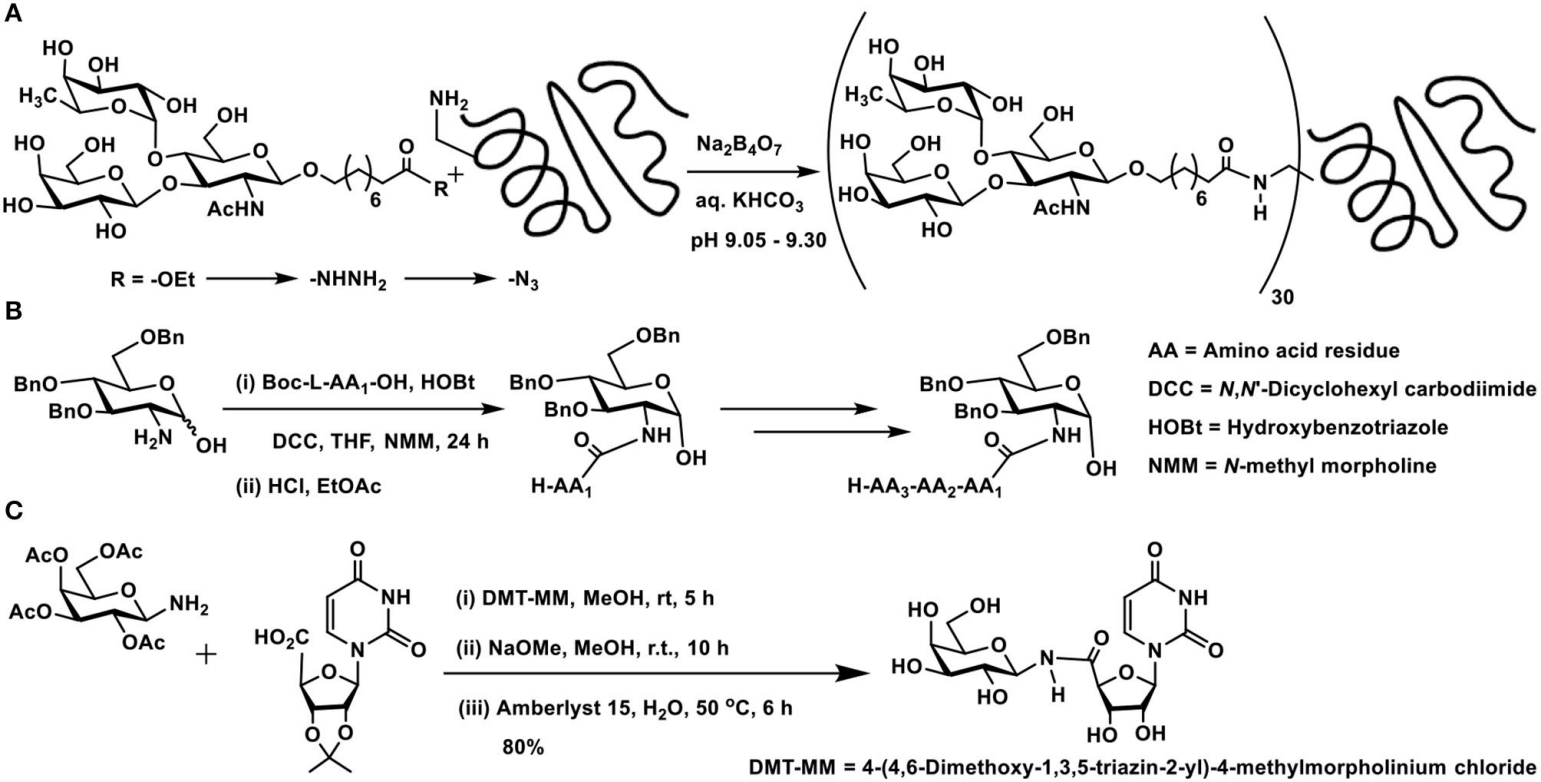
Amidation of: **(A)** a trisaccharide with BSA protein; **(B)** 2-amino-2-deoxy sugar with amino acids; **(C)** carboxylic acid derivative of a nucleobase using a glycosyl amine.

In order to retain the reducing end sugar in the cyclic form, tethers that present either an amine or a carboxylic acid moiety are preferred. Lemieux et al. developed the linker-assisted glycoconjugations through amide linkage. 9-Hydroxynonanoate ester was utilized as aglycon, the glycosylation of which with chosen sugar moieties was conducted initially. The ester functionality was taken through steps of (i) hydrazide formation; (ii) acyl azide formation; and (iii) reaction of the acyl azide with protein, in this instance, BSA protein (Scheme 1A). The extent of substitution of 30–38 ligands per protein was variable, depending on the sugar ligands whether mono-, di-, and trisaccharide (Lemieux et al., 1975).

Whereas, the acyl azide method is efficient to react with amines in proteins, the reaction is to be conducted under basic conditions, in order to prevent accumulation of HN_3_. All available lysines react without a site selectivity. Curtius rearrangement is an impediment in this reaction, formation of isocyanate and reaction with amines to form urea linkages are to be avoided. Yet the reaction is attractive, as hydrazide is formed directly from the ester and the reaction of acyl azide with protein occurs at a low temperature, under mild conditions that are suitable to retain configurations of chiral centers.

In place of antigen conjugation to BSA, conjugation to human serum albumin (HSA) was performed, in an effort to enhance the serology tests for mycobacterial disease-causing pathogens. Semi-synthetic trisaccharide, possessing a propionic acid ester was taken through the hydrazide, acyl azide formations, and coupling to HSA. The conjugation afforded 33.7 molecules of the antigen covalently conjugated to one protein (Moura et al., 2014).

Unprotected sugars, having the lactol at the reducing end, undergo oxidation to the corresponding lactone, the *in situ* reaction of which with amines leads to form the amides. Mono-, di-, and tri-saccharides were converted to the amides in one-pot, thereby opening up a method for a direct amidation of lactols in one pot. α-Keto acids also undergo amidation, through a decarboxylative amidation, promoted by iodine, as in the case of sialic acid, Kdo, and other α-keto acids (Cho et al., 2009). Presence of a carboxylic acid at other sites within the molecule does not interfere. Lysine containing peptides also underwent amidation involving side chain amine moiety.

2-Amino-2-deoxy sugars provide a good handle to conduct amidation with carboxylic acids under standard amidation protocol, involving activation of the acid using carbodiimides. Amine-protected amino acids were used in order to form amides with 2-amino-2-deoxy sugars (Zhang et al., 2011). The lactol moiety at the anomeric carbon of such sugars remained intact during the reaction. Up to tetrapeptides were utilized to form amide bond with the amine moiety at *C*-2 carbon of the sugar lactol (Scheme 1B).

Glycoconjugation of nucleobase, in which the phosphoester replaced with an amide, has been developed as potential glycosyl transferase inhibitors (Pastuch-Gawolek et al., 2017). Carboxylic acid, which formed the *C*-5 carbon of ribose moiety of the nucleobase, was activated to an activated ester, followed by reaction with glycosylamine (Scheme 1C). 1,4-Disubstituted 1,2,3-triazole linker between the glycopyranosyl and nucleobase derivative was also installed.

Among amide-based coupling, utilizing 3,4-dialkoxycyclobut-3-ene-1,2-dione, namely, squaric acid dialkylesters, has become a successful method of conjugation (Tietze et al., 1991; Wurm and Klok, 2013). Several dialkyl esters of squaric acid were utilized in the reaction, including those diesters that are soluble in water. Whereas, the first amidation reaction occurs easily with the use of excess squarate diester, the second amidation requires alkaline pH for amidation. Higher pH might tend to hydrolyze the monoester, thus a higher reactivity of the amine is important for the completion of the second amidation. Steric hindrance to reactive moiety renders a slower reaction rate, thus mono- and smaller oligosaccharides undergo faster amidation reaction than larger oligosaccharides.

**Scheme 2:**
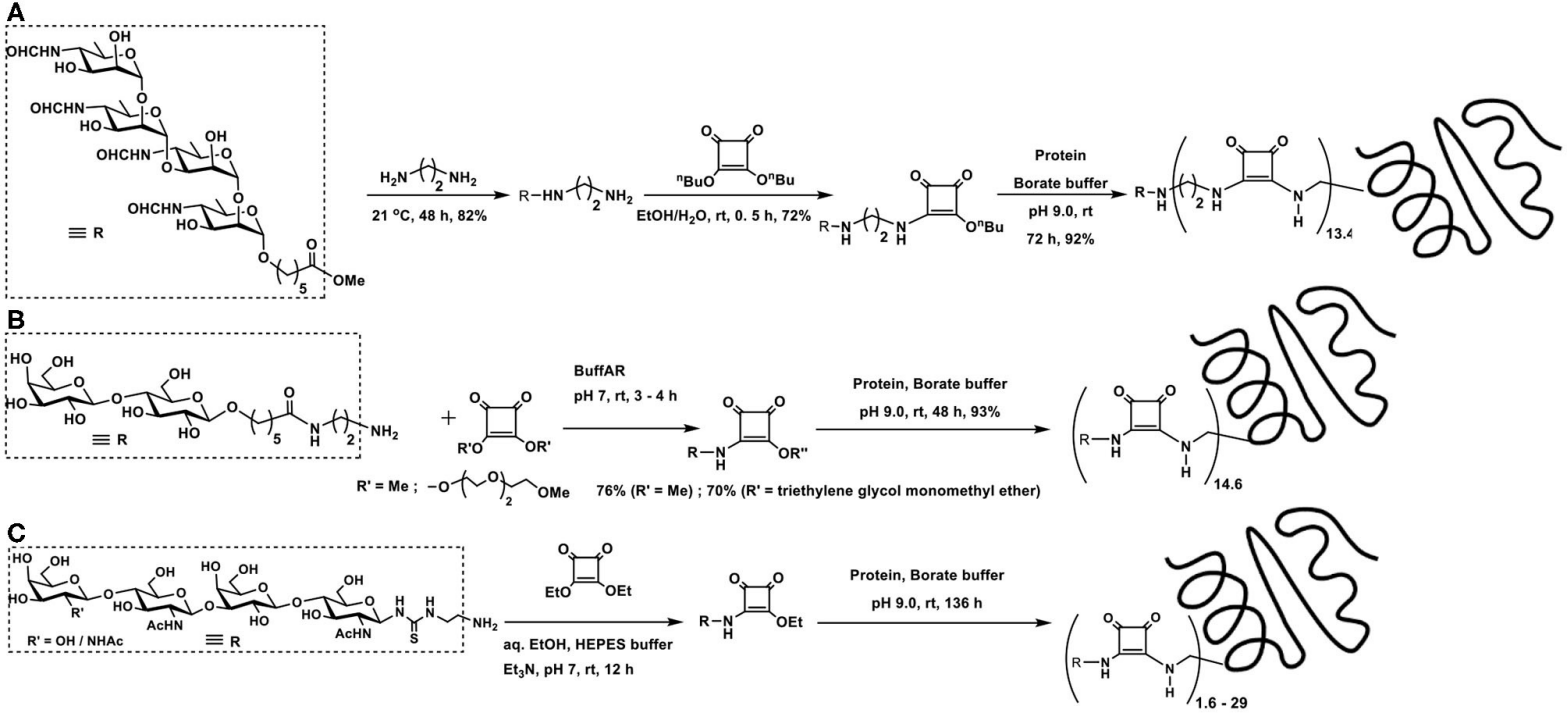
Glycoconjugation of: **(A)** a *Brucella* cell wall component tetrasaccharide with BSA using bis-squaramide; **(B)** two types of squarate diesters; **(C)** LacNAc-containing tetrasaccharide on to BSA.

Two types of polysaccharide sequences are present in one molecule of pathogenic *Brucella* species cell wall component. In an effort to understand the molecular recognition properties with antibodies of infected hosts, synthetic oligosaccharides, di- to hexa-saccharides, possessing both types of glycosidic linkages as in the native polysaccharides, were synthesized and studied by Bundle and co-workers (Ganesh et al., 2014). 5-Methoxycarbonylpentyl glycosides were taken for further elaboration of reactions with (i) 1,2-diaminoethane; (ii) dibutyl squaric acid diester; and (iii) protein BSA and good yields of target products were obtained in these reactions (Scheme 2A).

A comparison of the amidation of squaric acid diesters, namely, a water-soluble di(triethyleneglycol monomethyl ether)squarate and dimethyl squarate was conducted in the glycoconjugation of amine-tethered lactosyl moiety to BSA (Scheme 2B). An examination of the nature of these two squarates in relation to the efficiency of hydrolysis, amidation with the glycoside component and subsequent amidation with protein BSA revealed that both these squarates underwent comparable rates of amidations and also hydrolysis reactions. The water-soluble squarate did not show much difference in the reactivity, as compared to that arising from dimethyl squarate (Xu et al., 2018).

In the recognition of events arising from carbohydrate-protein interactions, multivalent ligand presentation of the epitopes is an important factor (Jayaraman, 2009). Multivalent ligand presentation aids to overcome weak binding potencies, the outcome of which has greater relevance in inhibitors design. Chemoenzymatic synthesis of LacNAc containing di-, tri-, and tetrasaccharides and conjugation on to BSA were conducted at sugar varying densities (Böcker et al., 2015). Squaric acid monoamides were formed initially by reaction of the squaric acid diethyl ester with the oligosaccharides in aq. EtOH, in the presence of Et_3_N, at room temperature overnight. With the excess use of the diester, efficient conjugation was ensured. Having the monoamides, the second amidation was conducted with BSA, at pH 9 for over 5 days, at differing molar ratios (Scheme 2C). These multivalent glycoconjugates were shown to possess varying binding potencies to the high affinity lectins, namely, galactin-1 and 3.

### Reductive Amination

Among methods developed for glycoconjugations, one of the early methods is the reductive amination, which consists in the reaction of a carbonyl functionality with an amine, followed by treatment of the intermediate imine with a hydride source. The reaction occurs in aqueous solutions (pH ~6–9), the water-soluble hydride source sodium cyanoborohydride, a mild reducing agent is used commonly to reduce imine to amine at room temperature. The target amine site in proteins is the side chain ε-amine site of lysine to form the intermediate imine derivative. Further, the reaction does not occur at most other amino acid side chain functionalities in a protein. However, in the case of the direct conjugation of sugars with proteins, introducing a linker connecting the sugars with proteins is practiced using bi-functional linkers. Conjugation through reductive amination is also practiced at the industrial production levels. The hydrolytic stability, covalent linkage, and facile coupling are among few features for the frequent utility of this conjugation.

**Scheme 3:**
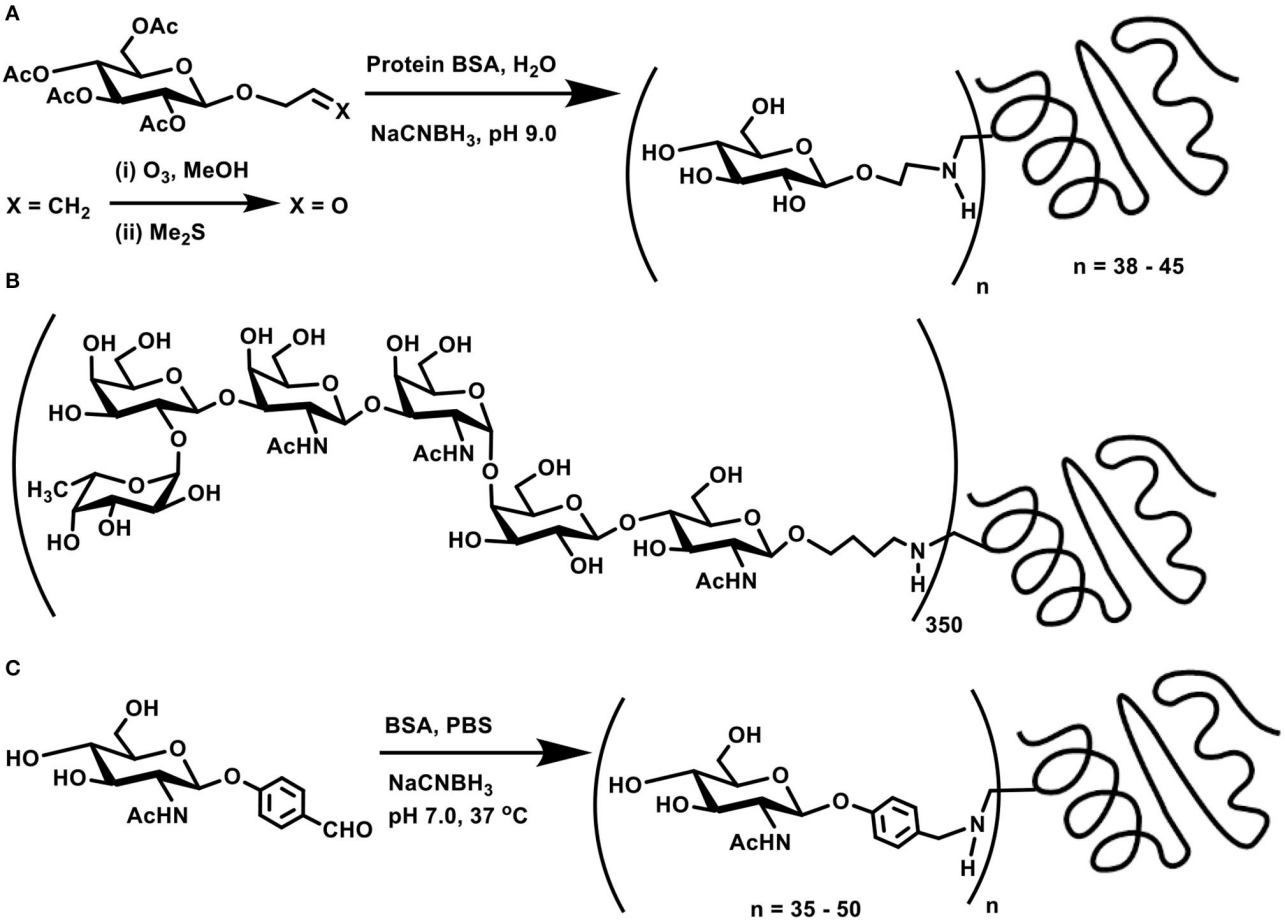
**(A)** Allyl glycoside as a precursor for ozonolysis and conjugation with BSA through reductive amination. **(B)** Molecular structure of MBr1 (globo-H) hexasaccharide-KLH glycoconjugate. **(C)** Reductive amination of protein BSA using *p*-formylphenyl glycoside.

Concerning the direct coupling of amines to sugar moieties, three approaches are adopted, namely, (i) the reaction of anomeric carbonyl function with chosen amines, followed by a reduction; (ii) generation of internal aldehydes through oxidation of vicinal diols to dialdehydes; and (iii) oxidation of the primary hydroxy moiety in sugars to the corresponding aldehyde, suitable for further reductive amination. In addition, bifunctional linkers are used, such that the conjugation occurs through the linker connecting the two functional moieties. Olefin functionality in linkers is made beneficial through ozonolysis and form aldehyde for further conjugation. One of the early methods known on reductive amination is the ozonolysis of allyl glycosides to the corresponding aldehyde product, which is then subjected to reductive amination on to a protein to afford the glycoconjugate product (Scheme 3A) (Bernstein and Hall, 1980).

Target oligosaccharide synthesis by chemical methods, followed by conjugation of the oligosaccharide with proteins, involves a number of considerations in the synthesis. Retaining the olefin functionality in the tether is preferred, as ozonolysis occurs smoothly to aid subsequent reductive amination. In this perspective, n-pentenyl moiety was utilized at the reducing end of a target oligosaccharide, namely, MBr1 (globo-H), a hexasaccharide antigen competent to induce antibodies against cancer cells. Ozonolysis of pent-1-ene moiety and a reductive amination of the resulting aldehyde with keyhole limpet hemocyanin (KLH) carrier protein led to realize the glycoconjugate vaccine candidate (Scheme 3B), with ~350 as the estimated number of epitopes attached to each molecule of the carrier protein (Allen et al., 2000). The hexasaccharide presented with an allyl moiety as the tether at the reducing end was also taken through ozonolysis and a subsequent conjugation to KLH through reductive amination, which led to a conjugation of ~150 oligosaccharides per protein (Ragupathi et al., 1997).

The reaction occurs easily with aromatic aldehydes tethered at the reducing end of the sugar moiety. Phenyl glycosides bearing *p*-formyl moiety at the phenyl substituent underwent a direct reductive amination with BSA, when conducted using NaCNBH_3_, in PBS buffer (pH 7) at 37°C. Time course analysis of the conjugation revealed a graded conjugation occurring at varying time intervals. Thus, aromatic aldehyde bearing *N*-acetylglucosamine moiety coupled covalently on to the protein, leading to 35, 43, and 50 lysine residues modification after 1, 2, and 4 days of reaction duration, respectively, as adjudged through amino acid analyses (Scheme 3C) (Roy et al., 1991).

**Scheme 4:**
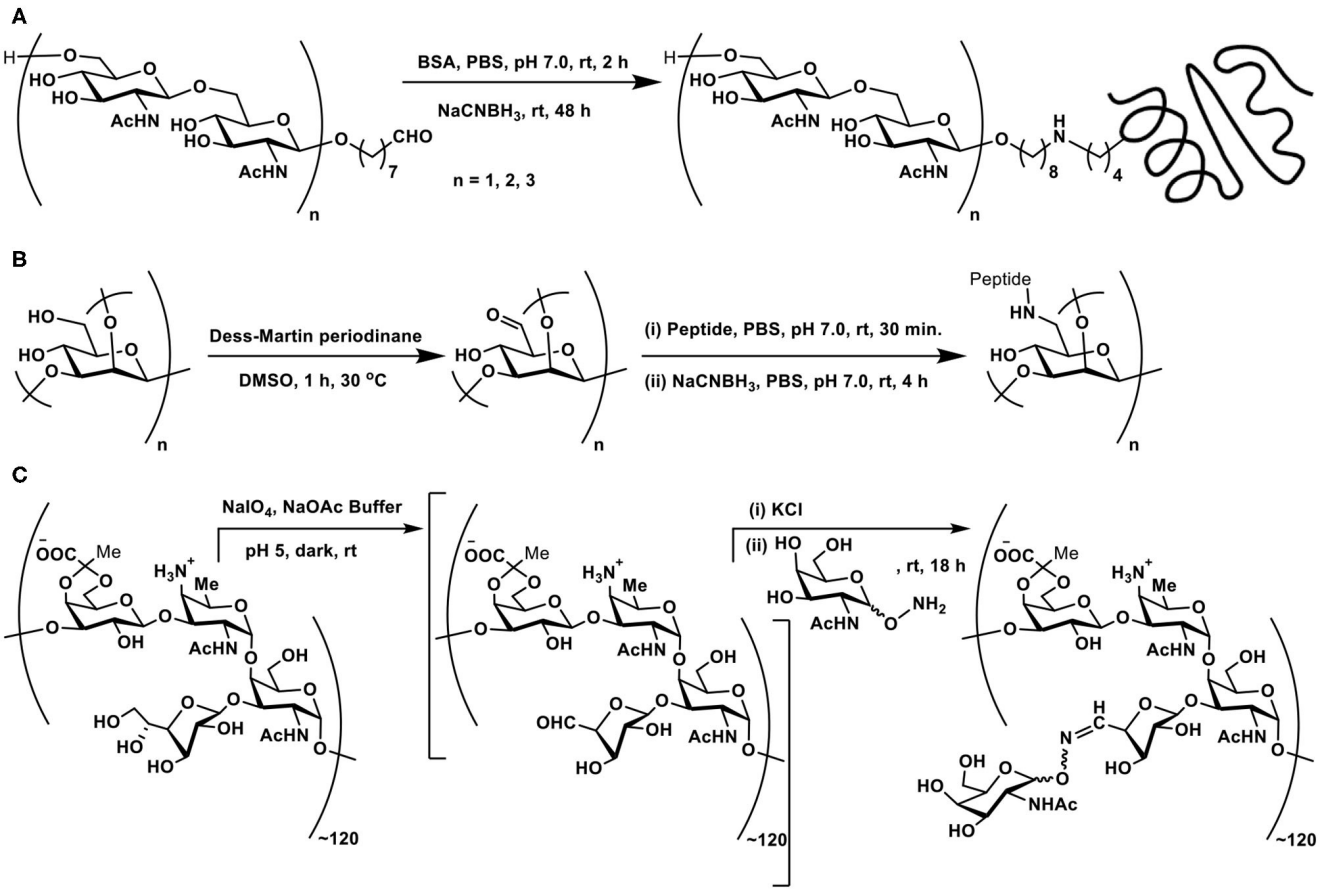
**(A)** Reductive amination of linker-tethered *N*-acetylglucosamine oligomers to BSA. **(B)** Oxidation of primary hydroxy groups in a mannan polysaccharide and subsequent conjugation with a peptide through reductive amination. **(C)** Zwitterionic polysaccharide conjugation with Tn-based glycosyl hydroxyamine.

The facile nature of conjugation allows different chain lengths of the sugar segment. Poly[β-D-(1 → 6)-*N*-acetylglucosamine] (PNAG) forms as a constituent of biofilms of *S. aureus* and *S. epidermis* bacterial pathogens grown on surfaces such as medical devices, for which conjugate vaccines provide a promise to completely eliminate biofilm grown pathogens. Chemical synthesis of di-, tetra-, and hexasaccharides, tethered with C7 alkylene chain terminated with an aldehyde moiety was followed further by reductive amination on to BSA. Here too, varying molar ratios of the sugars were utilized to secure glycoconjugates possessing varying sugar densities conjugated to the protein (Scheme 4A) (Fekete et al., 2014).

The primary hydroxy group at C-6 carbon can be oxidized selectively to the aldehyde functionality, which, in turn, can be subjected to reductive amination. This approach was demonstrated involving a branched mannan polysaccharide, having β-(1 → 2), α-(1 → 2), and α-(1 → 3) branchings. The mannan polysaccharide, with ~35 kDa molecular mass and polydispersity index of 1.6, was subjected to Dess-Martin periodinane reagent-based oxidation, by which the primary hydroxy moiety at C-6 carbon oxidized to aldehyde. Further, peptide coupling with the newly-formed aldehyde under reductive amination condition was adopted to secure mannan-peptide conjugates. The 14 amino acid residue peptide was further incorporated with lysyl-lysine dipeptide so as to increase conjugation sites in the peptide. The peptide content in the conjugate was estimated to be 28 ± 5 mass % or 3 ± 0.5 mol% (Scheme 4B) (Farkaš et al., 2016).

A zwitterionic polysaccharide as a powerful carrier for glycoconjugations was demonstrated by Andreana et al. The identified polymer was a fermentation product of colon bacterium *Bacterioides fragilis*, possessing a zwitterionic tetrasaccharide repeating unit. The vicinal diol moieties provided a tool for oxidative cleavage. Thus, a controlled periodate-mediated oxidative cleavage of exocyclic diol moiety to an aldehyde, followed by reaction with an amine afforded the zwitterionic polymer. In this instance, Tn antigen-based glycosyl hydroxy amine afforded the corresponding oxime linkage (Scheme 4C) (De Silva et al., 2009).

**Scheme 5:**
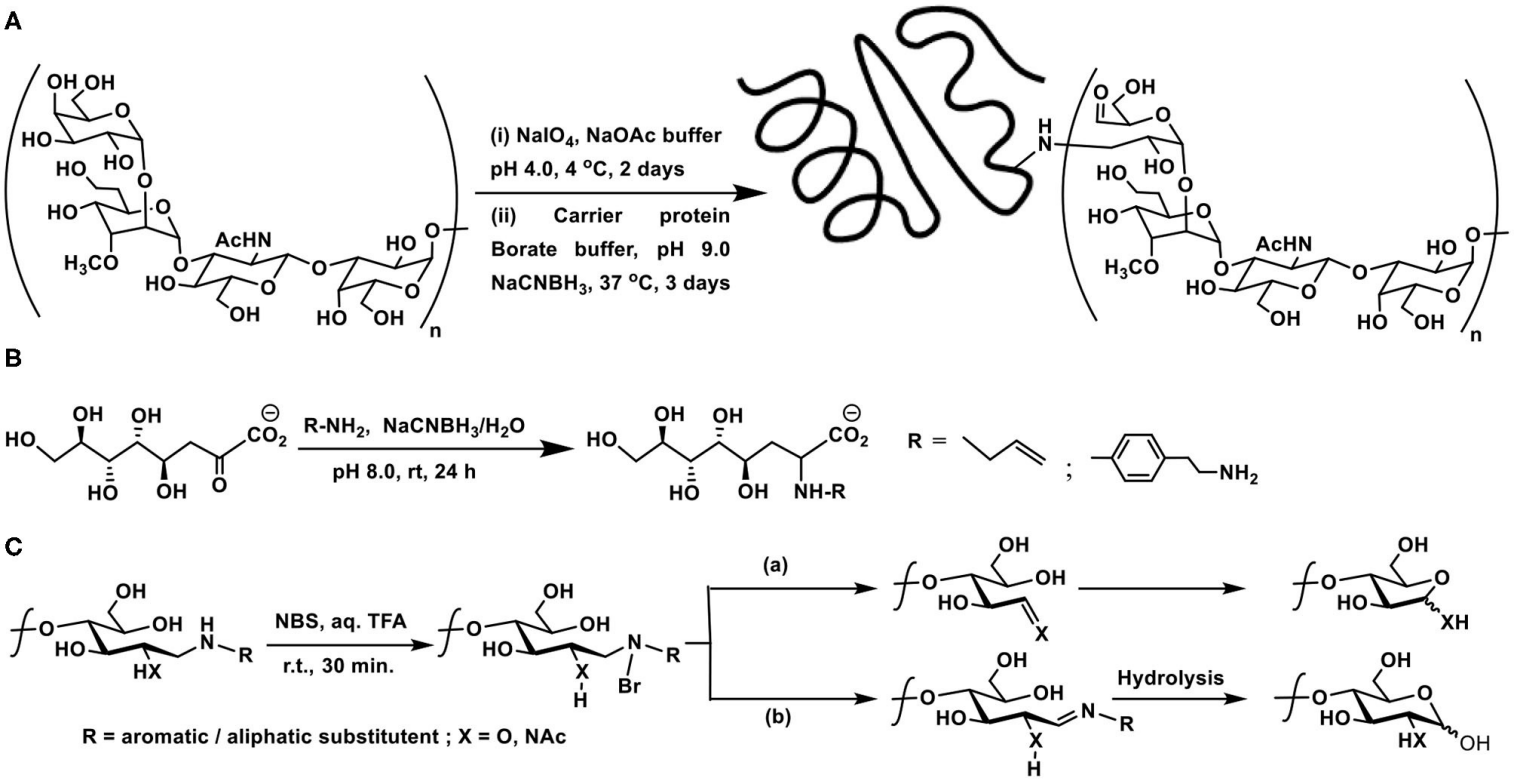
**(A)** Oxidative cleavage at the non-reducing end of capsular polysaccharide CPS of *Campylobacter jejuni*, followed by reductive amination with carrier protein diphtheria toxin mutant CRM197. **(B)** Reductive amination of Kdo by allylamine and 2-(4-aminophenyl)ethylamine. **(C)** NBS-mediated cleavage of the product of reductive amination. Pathways **(a)** or **(b)** are dependent on the nature of the substituent R.

The non-reducing end of a polysaccharide offers vicinal hydroxy groups which are amenable for oxidative cleavage to afford aldehyde moieties. Many enteric pathogens possess unusual surface polysaccharides called capsular polysaccharides (CPS). CPS conjugate vaccines were developed as potent vaccines against such enteric pathogens. Isolation of CPS from bacterial cells, periodate-mediated oxidation of sugar moieties at the non-reducing end of the polysaccharides, with an average molecular mass of ~5,500–6,500 Da, followed by reductive amination-based conjugation to a carrier protein, namely, diphtheria toxin mutant CRM197, was demonstrated (Scheme 5A) (Monteiro et al., 2009). Conjugation with the carrier protein afforded 2–5 residues of CPS per carrier protein, as adjudged through SDS-PAGE analysis.

Direct reductive aminations of ketoses are inefficient as compared to aldoses at the reducing end of oligo- and polysaccharides (Kubler-Kielb, 2011). The major bacterial outer membrane lipopolysaccharide constituent possesses−3-deoxy-D-*manno*-octulosonic acid (Kdo), which is a ketose, in the core region. Kdo, devoid of the fatty acid portion, is obtained through mild acid hydrolysis of the lipopolysaccharide and is used for the polysaccharide conjugations. Reductive amination with chosen linkers was explored earlier on Kdo molecule. Reaction of Kdo with allylamine and 2-(4-aminophenyl)ethylamine under reductive amination conditions was conducted, in order to assess the effectiveness of the reaction on this keto-acid. Reductive amination occurs faster on aldoses than keto-acids generally. The yields of ~50–60% of the reductive amination product did not differ much with temperature, though Kdo products with reduction of ketone functionality also formed, without the desired reductive amination, as a result of Kdo undergoing formation of lactone, anhydro derivatives, upon varying the reaction duration, temperature and concentrations. The reaction also highlighted aromatic amine reacting more favorably than aliphatic amine, in the case of 2-(4-aminophenyl)ethylamine, as a result of the reaction conditions adopted herein (Scheme 5B) (Kallin et al., 1986; Grimmecke and Brade, 1998).

The reaction of aromatic amines undergoing facile conjugations with the reducing sugars brings in another important discovery of reverting the newly formed C-N bond back to amine and aldehyde/ketone. The possibility of cleaving the newly formed amine bond with the aid of an electrophilic reagent was developed. *N*-Bromosuccinimide activates the cleavage reaction of the newly-formed amine bond. Upon formation of the N-Br bond, a sequence of rearrangement occurs, leading to formation of either an imine or one-carbon reduction product through a C-C bond cleavage. These intermediates lead to either a pyranose or a furanose, with the loss of the primary amine which is utilized initially to conduct the reductive amination (Scheme 5C). The furanose product formed as the major product with most substituted aromatic amines. An aliphatic amine was also used, in this instance, 1,2-diaminoethane was used to conduct the reductive amination, so as to secure mono-amine glycoconjugate. NBS-mediated cleavage of this mono-amine glycoconjugate afforded pyranose as the major product (Song et al., 2013). The report emphasizes the use of aromatic amines as a resourceful cleavable linkers for glycoconjugations.

### Native Chemical Ligation

**Scheme 6:**
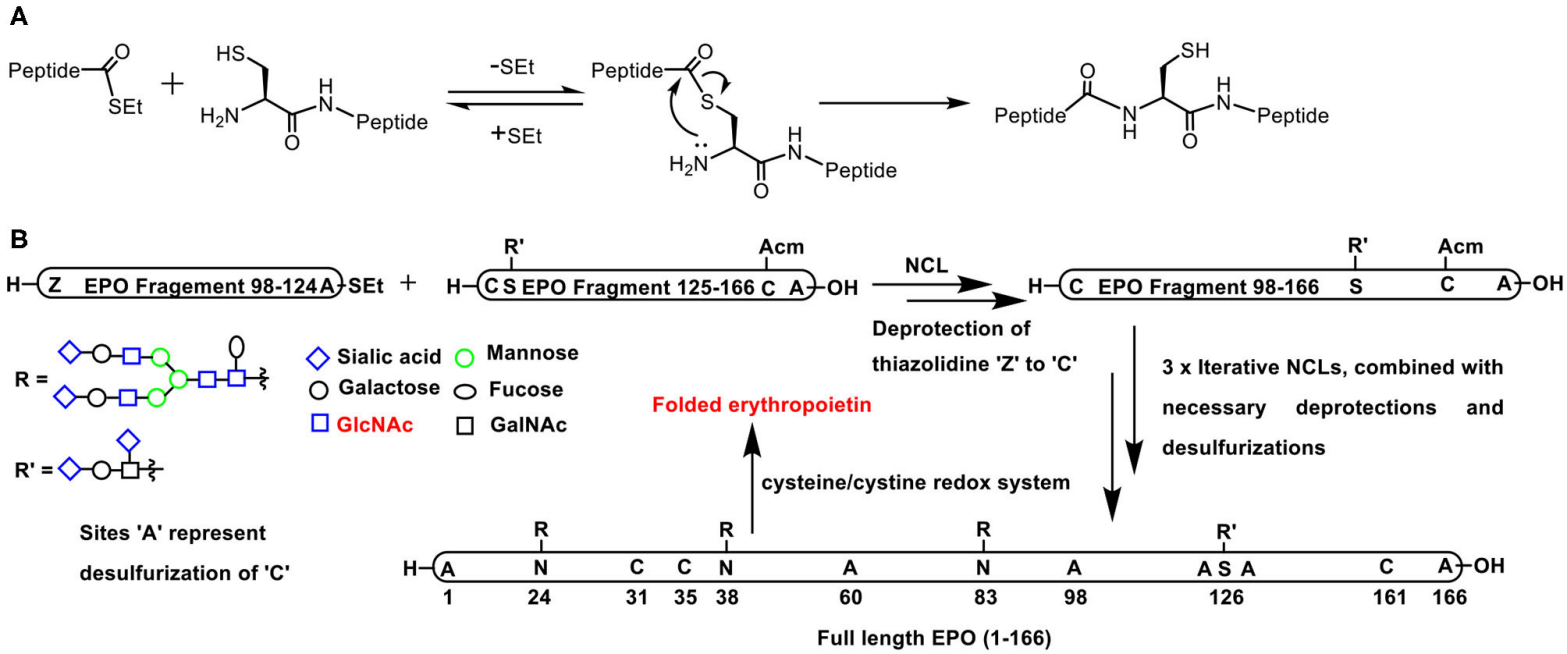
**(A)** Native chemical ligation involving the reaction of a *C*-terminus thioester with *N*-terminus cysteine to afford a new peptide bond retained with a free cysteine. **(B)** Full length erythropoietin synthesis involving *C*-terminus thioester and *N*-terminus cysteine in EPO fragment pairs (i) 98–124 and 125–166; (ii) 60–97 and 98–166; (iii) 29–59 and 60–166; (iv) 1–28 and 29–166, respectively, undergoing NCL, combined with necessary deprotections, desulfurizations, and the final step of folding through cysteine-cystine redox reaction.

Native chemical ligation (NCL), introduced and adopted very effectively to prepare oligo- and polypeptides, pertains to the reaction of a thioester functionality with a free thiol to form a new thioester. In the presence of a proximal amine functionality, as in the case of *N*-terminal cysteine residue, an *S*-*N* acyl migration occurs, to afford an amide product possessing a free thiol moiety (Scheme 6A) (Dawson et al., 1994).

Pertaining to glycoconjugations, conjugation of glycopeptide moiety with yet another peptide or glycopeptide is conducted so as to achieve the synthesis of target homogeneous glycopeptide in a convergent manner. An elegant application of the NCL is the synthesis of a 166 amino acid residue containing glycoprotein, namely, erythropoietin. With judicious application of NCLs consecutively in multiple steps, the smaller glycopeptide oligomers were transformed to medium size oligomers and finally to the large target polypeptide. A tight control of the amino acid residue undergoing the glycoconjugation was achieved as a result of controlled synthesis of smaller fragments that were put together subsequently in a highly convergent fashion (Scheme 6B) (Wang P. et al., 2013).

**Scheme 7:**
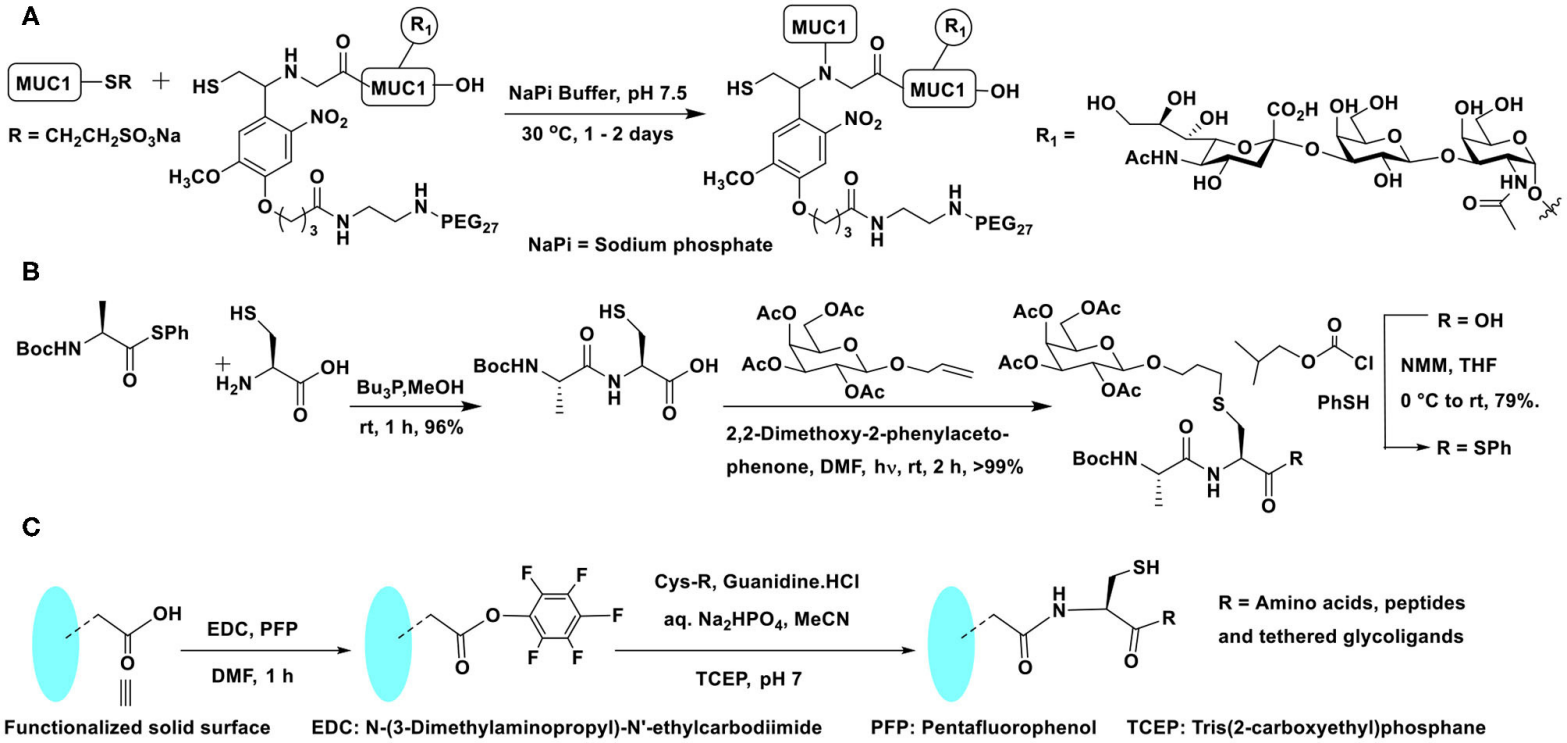
**(A)** NCL on mucin 1 peptide and photocleavable auxiliary containing glycopeptide fragments to afford defined mucin 1 glycopeptides. **(B)** NCL and thiol-ene coupling to prepare a glycopeptide. **(C)** Oxo-ester activation to an *in situ* generation of thioester intermediate and subsequent NCL reaction on a functionalized solid surface.

Conjugation of glycopeptide fragments of *O*-glycosylated mucin-1 was demonstrated, so as to secure polypeptides presented with chosen glycosides in the glycopeptide fragment precursors (Scheme 7A). Further novelties of the glycopeptide fragment include the installation of pegylated photolabile auxiliary to enable enzymatic glycosylation on to the peptide fragment and the preparation of required thioester of a glycopeptide fragment at the *C*-terminal from the corresponding hydrazide (Bello et al., 2015). Glycopeptide fragments subjected NCL to form large homogeneous glycopeptides are practiced increasingly (Du et al., 2016).

A combination of conjugations was used to secure glycoconjugated peptides. The NCL enabled the conjugation of different parts of the peptides and the glycoconjugation step was performed with the aid of thiol-ene coupling (TEC). The repetitive cycles of NCL and TEC beginning with coupling of protected alanine thioester and free cysteine afforded Ala-Cys dipeptide. Dipeptide, in turn, was reacted with protected allyl glycoside to furnish glycoconjugated dipeptide. The *C*-terminal carboxylic acid was then converted to an active thioester, equipping the dipeptide ready for a subsequent cycle of NCL (Scheme 7B) (Markey et al., 2013).

In order to overcome the protection of group compatibilities and enable a facile NCL on surfaces, Flitsch et al. developed a pentafluorophenyl ester activation at the *C*-terminal carboxylic acid moiety. A pentafluorophenyl ester activation at the surface was found to be optimal and the reaction occurred selectively with cysteine, in the presence of several other amino acids. The activated pentafluorophenyl ester led to thioester formation upon reaction with cysteine favorably, as a result of the faster *O* → *S*-acyl transfer process. The *in situ* formed thioester underwent NCL reaction and afforded the amide bond (Scheme 7C). Control experiments demonstrated the formation of the thioester intermediate, thereby reiterating faster reaction with cysteine, as compared to many other amino acids (Weissenborn et al., 2012).

### Staudinger Ligation-Based Glycoconjugations

Reaction of an azide with trivalent phosphorus to afford iminophosphorane or aza-ylide is referred to as a Staudinger reaction. Subsequent hydrolysis of the iminophosphorane intermediate provides an amine. On the other hand, the reaction of iminophosphorane adduct with acylating agents leads to the formation of amides. Numerous applications of this reaction are known, included further with the facile nature of the reaction under biological milieu. As a result of the latter application, bio-orthogonality of the reaction has been carefully developed, leading to the reaction being an established method for bioconjugations (Bednarek et al., 2020). The methods of acylations determine whether the pentavalent phosphorus is retained in the reaction product or is removed at the end of the reaction. These methods are referred to as non-traceless and traceless Staudinger reactions, respectively.

**Scheme 8:**
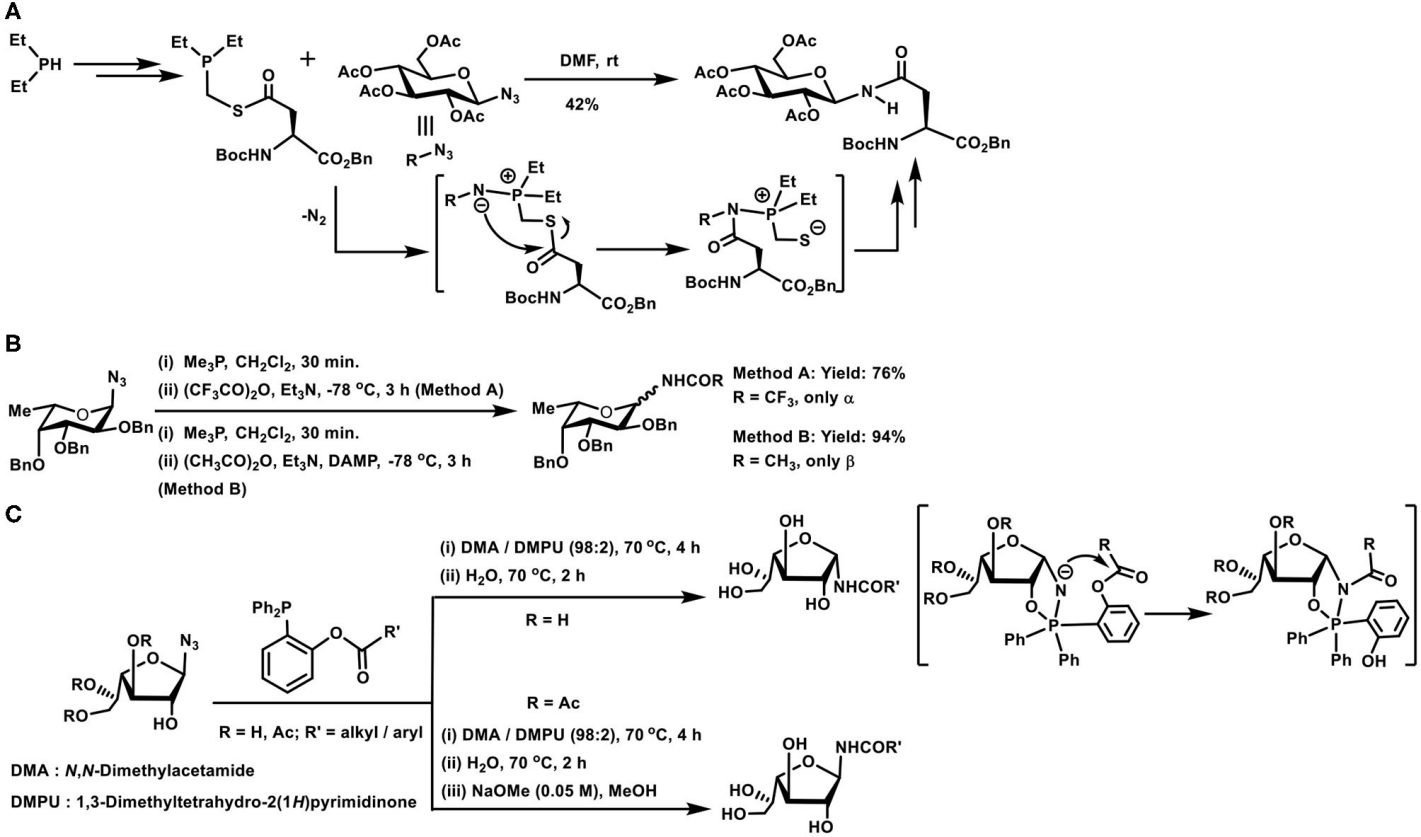
**(A)** Synthesis of an *N*-glycosylated amino acid derivative through the Staudinger ligation. **(B)** Glycosyl amides formation with the aid of Staudinger reaction. **(C)** Traceless Staudinger reaction to prepare *N*-glycosyl amides. Key intermediates of the reaction are given in the bracket.

Glycosyl azides provided the earliest possibility to implement the Staudinger reaction with sugar components. When aspartic acid was involved, the side chain carboxylic acid was derivatized to an *N*-glycosyl amide. Such an amide would correspond to the side chain *N*-glycosylated asparagine derivative (Scheme 8A). Key to the reaction is the preparation of the corresponding phosphinothioester, initiated from dialkyl/arylphosphane through a sequence of transformations. A facile Staudinger reaction occurred in DMF and *N*-glycosyl amide containing aspargine derivative was secured. The reaction afforded the β-anomer conjugation product, irrespective of the glycosyl azide was α- or β-anomer (He et al., 2004).

Reaction of azides leading to amides in one step, with the aid of trivalent phosphorus reagents, find an importance in the preparation of glycosyl amides, subjected to anomeric selectivities of the amide formation. Glycosyl azides undergo a Staudinger reaction, producing the iminophosphorane in the first step, followed by reaction with an acylating agent to form glycosyl amides (Scheme 8B). Iminophosphorane intermediate undergoing anomerization is a limiting factor under the conditions, highly reactive trifluoroacetic anhydride provided the α-anomer, whereas milder acetic anhydride led to β-anomer product, as a result of the anomerization. Efforts also showed that a functionalized phosphane, having 2-acetoxy substituent in one of the phenyl substituents in triphenylphosphane, mediated the reaction and the acetamide product was secured with major α-selectivity (Bianchi and Bernardi, 2004).

The role of the protecting group at *C*-2 hydroxy moiety and the configuration also dictates the course of the reaction leading to the formation of *N*-glycosyl amides, particularly when the reaction is performed under traceless reagent condition (Scheme 8C). In the absence of the protecting group and leaving the hydroxy functionality free at *C*-2 carbon, the reaction occurs to afford the product in α-anomeric configuration (1,2-*cis*-orientation). Whereas, an ester protecting group at *C*-2 leads to the *N*-glycosyl amide product in the β-anomeric configuration (1,2-*trans*-orientation). Free hydroxy group in axial or equatorial orientation always provided the 1,2-*cis*-amide, and protected hydroxy group always provided the 1,2-*trans*-amide. A cyclic oxaphospholane intermediate formation, from an initial acyclic phosphinimine adduct, followed by the *O*- to *N*-acyl intramolecular transfer accounted the product formation with 1,2-*cis*-orientation, when the hydroxy group at *C*-2 was free (Nisic and Bernardi, 2011; Nisic et al., 2012).

**Scheme 9:**
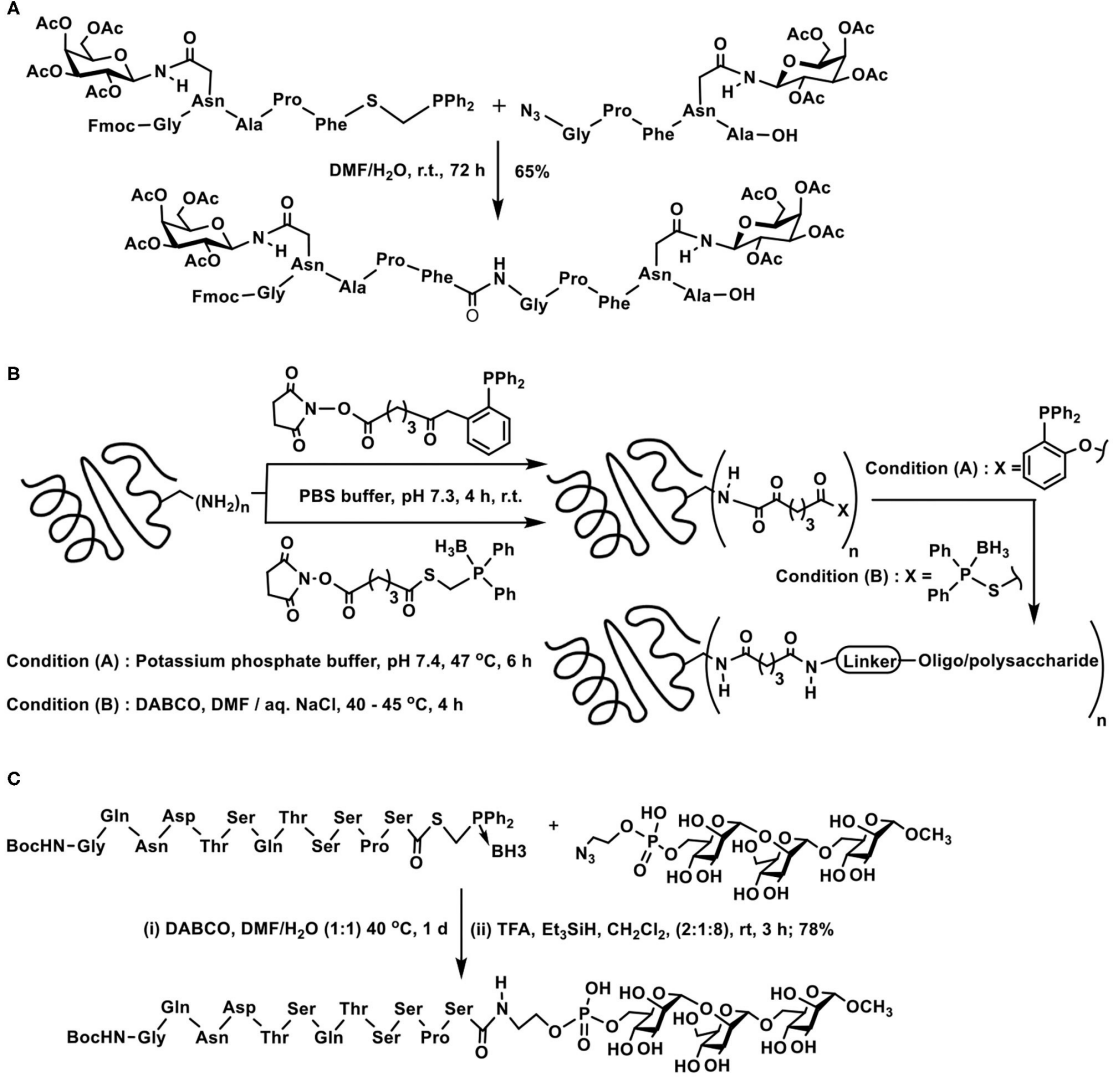
Staudinger ligation of: **(A)** two glycopeptide fragments to secure glyco-oligopeptide; **(B)** oligo/polypeptides on to carrier proteins tetanus taxoid and bovine serum albumin; **(C)** a peptide fragment with azidoethylphosphate-derivatized mannopyranosyl trisaccharide.

A first instance of a glycopeptide synthesis with the aid of traceless Staudinger reaction was demonstrated by Wong and co-workers. In the traceless Staudinger ligation, the azido-containing peptide fragment reacts with the *S*-diphenylphosphanomethyl thioester containing peptide fragment to afford the new peptide bond. The *C*-terminal end about the newly generated amide bond possesses peptide fragment of the azide reactant and *N*-terminal possesses the peptide fragment from the thioester reactant. Incorporation of *O*- and *N*-glycosyl-tethered peptide in either of these synthons permits new peptide bond formation, provided the glycoside-containing amino acid residue is located few residues away from the reactive site (Scheme 9A) (Liu et al., 2006).

Glycoconjugation of protein carriers with chosen glyco-epitopes was conducted efficiently with the aid of traceless Staudinger ligation. The ligation method was used effectively to conjugate haptens pertaining to lipopolysaccharide components of *S.flexneri* and *V*. *cholerae*, the haptens being either a pentasaccharide or a large branched polysaccharide. The approach was to conduct (i) amidation of available amines of the carrier protein with an activated carboxylic acid containing a distal diphenylphosphanophenyl ester or *S*-diphenylphosphanomethyl thioester and (ii) reaction of the resulting protein modified with the thioester moieties with azide tethered oligo- and polysaccharides (Scheme 9B). In order to prevent oxidation, phosphane moiety was protected as the borane complex, which required *in situ* decomplexation prior to the ligation reaction. Thus, the addition of base DABCO was required during ligation with *S*-diphenylphosphanomethyl thioester acyl donor component. The reactions afforded the glycoconjugated products in moderate yields, with ~3–7 moles of sugar *per* mole of the protein (Grandjean et al., 2005).

Staudinger ligation is demonstrated to be resourceful for the conjugation of peptides with glycosylphosphatidylinositol (GPI) fragments. Synthesis of a human CD52 antigen analog by coupling conserved trimannose moiety with complete CD52 peptide sequence was accomplished through Staudinger traceless ligation. The peptide was equipped as the acyl donor, whereas, phosphate functionalized glycoside, further coupled with an azide functionality acted as the acyl acceptor. *S*-Diphenylphosphanomethyl thioester, protected with borane at the phosphorus center, was identified for coupling with the peptide fragment. Whereas, the phosphate moiety tethered with azide moiety and further linked to a tri- and tetrasaccharide was chosen. Application of the traceless Staudinger ligation methodology led to the desired amide bond formation connecting the peptide fragment with the sugar fragment (Scheme 9C) (Zhu and Guo, 2017).

**Scheme 10:**
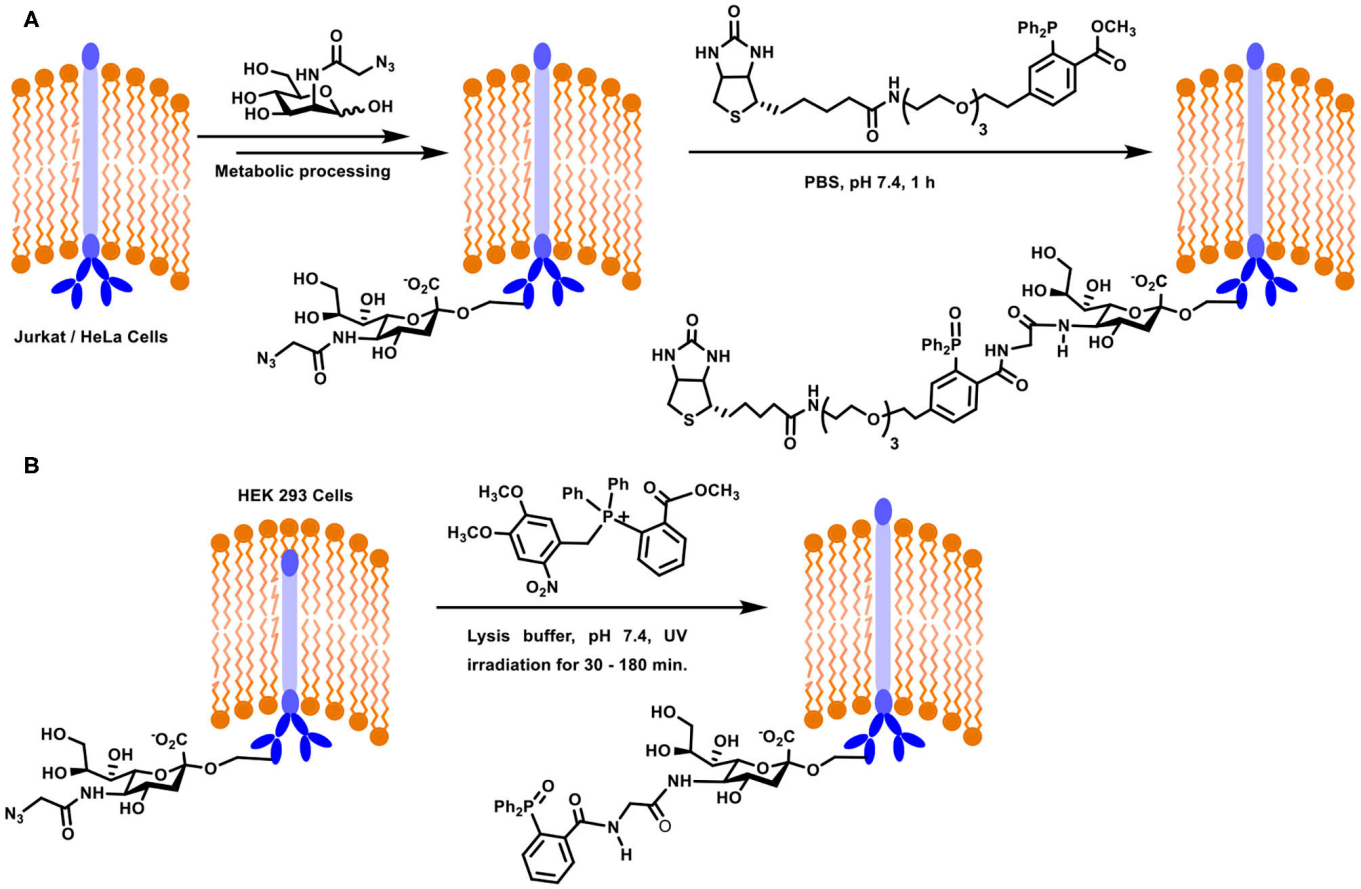
**(A)** Metabolic engineering and Staudinger ligation at the Jurkat / HeLa cell surfaces. **(B)** Staudinger ligation of sialic acid derivative on HEK293 cells using photocleavable precursor.

A large part of the contemporary developments on the Staudinger ligation method for the glycoconjugation of biological targets is accounted for by the seminal works of Bertozzi and co-workers. Metabolic engineering of cell surfaces, followed by the ligation provided the much-needed handle to tag chosen conjugates on to the cell surfaces. In an approach, the biosynthetic machinery precursor for sialic acid, namely, mannosamine, was taken advantage of to introduce azide-functionalized sialic acid at the cell surface. The approach required azide-functionalized mannosamine as the substrate for the biotransformation. *N*-Azidoacetylmannosamine was designed as the precursor for this purpose, the incubation of which with Jurkat and HeLa cells was taken up by the biosynthetic machinery. After 3 days of incubation, the azide-functionalised sialic acid was metabolically processed and expressed on the cell surface (Scheme 10A). The expression was analyzed using a biotin-labeled phosphane derivative, which underwent the Staudinger ligation in the biological milieu. The fluorescein isothiocyanate-avidin-biotin interaction was utilized further to follow the conjugation by fluorescence and flow cytometry. Further important observation was the growth rate of the cells, which was not affected as a result of the azide-incorporation in sialic acid, implying the compatibility of the substrate. Further, reduction of the disulfide bond in protein by phosphane did not occur. These experiments established the new method to introduce the chemically-reactive, biologically compatible azide functionality on to the cell surfaces and the ability to undergo desired ligation reactions (Saxon and Bertozzi, 2000). The reaction is referred to as a Staudinger-Bertozzi ligation, pertaining to the reaction occurring at the cell surfaces.

Oxidation of the phosphane reagent prior to the ligation reaction is a potential limitation in the above reaction. A novel approach to overcome this limitation was addressed recently (Shah et al., 2016). In this approach, a photolabile moiety was installed at the phosphane center, to secure a tetravalent phosphane. *In situ* generation of phosphane through light activation and subsequent reaction with the azide, introduced through azide-tethered mannosamine to sialic acid metabolic processing, was verified *in vitro* on mammalian cells and *in vivo* on live zebra fish. When the photo-release reaction was conducted in the presence of the azide component, ligation proceeded to afford target amide product. HEK293 cell lines were used in the study in order to evaluate the Staudinger ligation using photolabile-moiety attached phosphane reagent. The cell line was modified initially with azide-linked sialic acid derivative using metabolic incorporation of azidoacetylmannosamine. The derivatized cell surface was incubated with photolabile phosphane, irradiated with UV light which enabled release of the active phosphane reagent, which underwent ligation with azide, leading to non-traceless Staudinger product (Scheme 10B). In addition to being able to protect the phosphane from oxidation, this method provided further possibility to irradiate a defined location of the cell surface.

### Azide-Alkyne Click Reactions

The 1,3-dipolar cycloaddition reaction involving an azide and a terminal alkyne to afford a triazole moiety is termed prominently as a click reaction. The reaction is catalyzed by Cu(I), commonly generated *in situ* from Cu(II)/sodium ascorbate, in a number of solvents, including aqueous solutions and buffer solutions. Glycoconjugation is also a major beneficiary of this reaction, in order to secure varied types of glyco-conjugated products, limited only to the imagination of the practitioner. Immense demand on conjugation of sugars, oligo-, and polysaccharides to molecular and macromolecular scaffolds led to further developments, so as to include this cycloaddition reaction applicable in an entirely biological milieu. Restrictions arise relating to the use of metal catalyst to activate the reaction, in the absence of which the reaction might require elevated temperatures. Inducing strain in the alkyne component became a viable approach to its activation. Internal alkynes provide an easy access to induce strain in the system, further included with ability to incorporate substituents that would alter electronic influences in the alkyne system. These strained alkynes undergoing the cycloaddition with azides without a metal catalyst became to be known as a “copper-free click reaction.” The copper-free click reaction has been eloquently demonstrated in glycoconjugations, pertaining to this chemical reaction occurring at the biological cell surfaces.

Cycloaddition of terminal alkyne and azide has a regiochemical outcome on the newly-formed triazole product, with substituents at one of the carbon and nitrogen sites. These products relate to 1,4- and 1,5-disubstitution on 1,2,3-triazole heterocycle. Thermal stimulus to the reaction leads to formation of both these products, whereas Cu(I)-catalyzed reactions enable formation of 1,4-disubstitution as the major product (Rostovtsev et al., 2002; Tornøe et al., 2002).

**Scheme 11:**
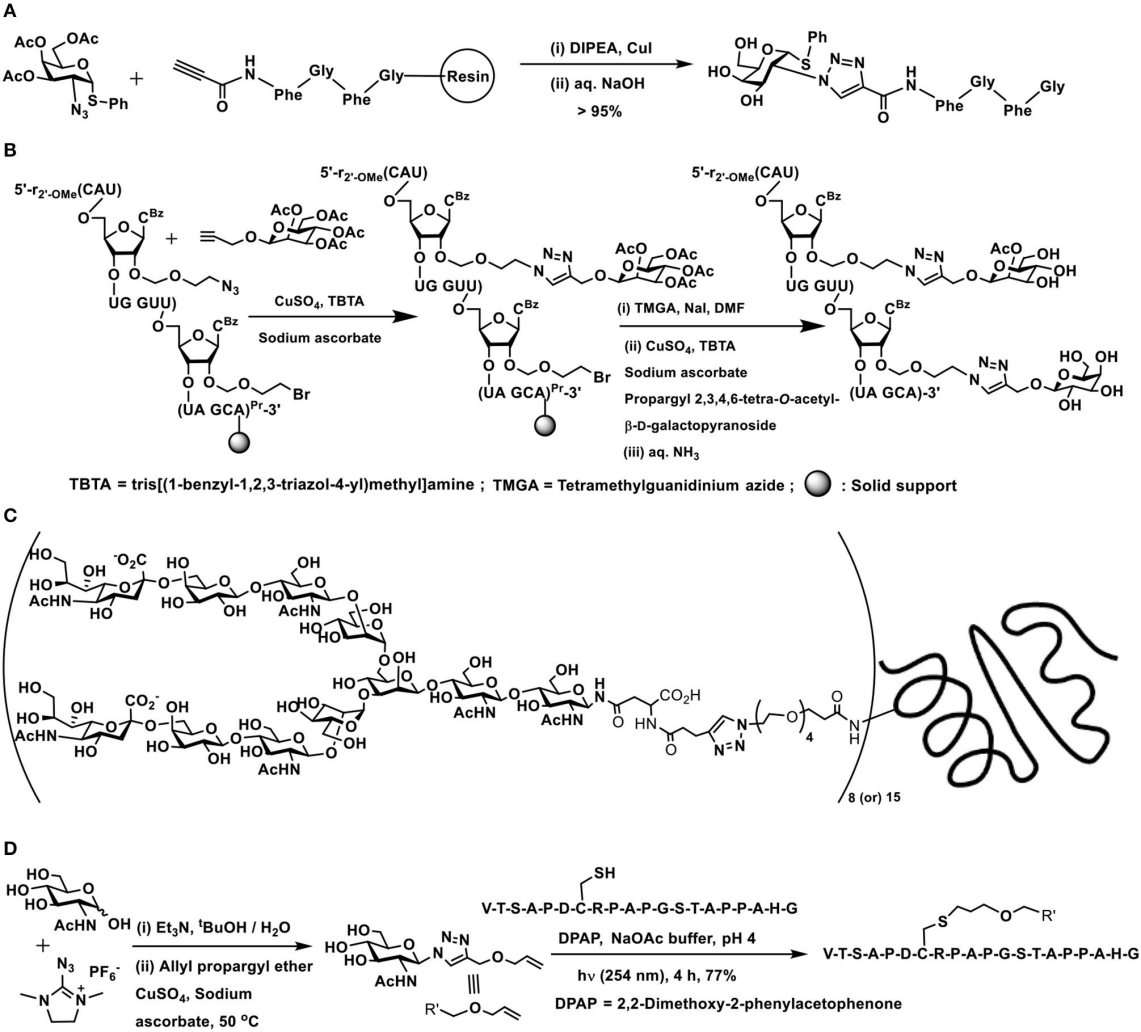
**(A)** Synthesis of a 1,2,3-triazole-linked 1,4-disubstituted glycopeptide conjugate. **(B)** A double click reaction at the *C*-2′ carbon of an oligonucleotide. **(C)** 1,4-Disubstituted [1,2,3]triazole linked complex biantennary *N*-glycan–HSA conjugate. **(D)** Double conjugation-based glycopeptide synthesis.

The early report of Meldal and co-workers forms the first instance of conjugation of a peptide with a monosaccharide. The tetrapeptide possessing an alkyne moiety at the amine terminal end and coupled to a solid phase resin at the carboxy-terminal was reacted with 2-azido thiogalactoside, in the presence of CuI/ *N,N*-diisopropylethylamine, afforded 1,4-disubstituted 1,2,3-triazole glycoconjugate (Scheme 11A) (Tornøe et al., 2002). Several elegant glycopeptides were reported incorporated with triazole linkers (Lee et al., 2012; Lim et al., 2014; Cheng et al., 2015).

Glycoconjugations with two differing glycosyl components in a growing oligonucleotide on the solid support in a step-wise manner was demonstrated. The approach was to incorporate the first propargyl glycoside with a pre-formed azide located in one of the nucleobases through the click reaction, followed by azidation and click reaction of another propargyl glycoside at yet another nucleobase (Scheme 11B). The oligonucleotide conjugates with two different sugars showed a small increase in double strand stability in a solution of low ionic strength, when it formed double strand with complementary DNA sequence (Kiviniemi et al., 2011).

The benign nature of the click reaction between terminal azide and alkyne finds an immediate applicability to generate multivalent glyco-ligands onto protein scaffolds. Biantennary undecasaccharide glyco-ligands were covalently linked on to the HSA protein scaffold. A complex asparagine-linked undecasaccharide possessed a Man-(1 → 3)-Man-(1 → 6)-Man trisaccharide core, having terminal sialic acid residues was constructed to study the efficacy of the multivalent ligands as antiviral and anticancer agents. HSA protein was installed with azide moiety, whereas the alkyne moiety was installed at the asparagine-residue of the oligosaccharide. Cu(I)-catalyzed click reaction was performed to enable the triazole formation and varying numbers of triazoles formed with the aid of differing molar ratios of the azide and alkyne (Scheme 11C) (Wang H. et al., 2013).

Methods are also developed involving two types of conjugations, namely, an azide-alkyne reaction and a thiol-ene reaction, as illustrated by Fairbanks and co-workers. A methodology for azidation of the anomeric carbon at the reducing end was devised, wherein unprotected free hydroxy groups containing sugars, carrying an acetamido moiety, were subjected to reaction with 2-azido-1,3-dimethylimidazolinium hexafluorophosphate in aqueous solution to afford the corresponding glycosyl azide in one-pot (Lim et al., 2014). Anomeric azide led to a reaction with an alkyne, in the presence of Cu(I), to form the 1,4-disubstituted triazole product. The presence of an allyloxy-tether in such a triazole product permitted a thiol-ene reaction, with the thiol moiety of cysteine present in an oligopeptide (Scheme 11D) (Alexander et al., 2018).

**Scheme 12:**
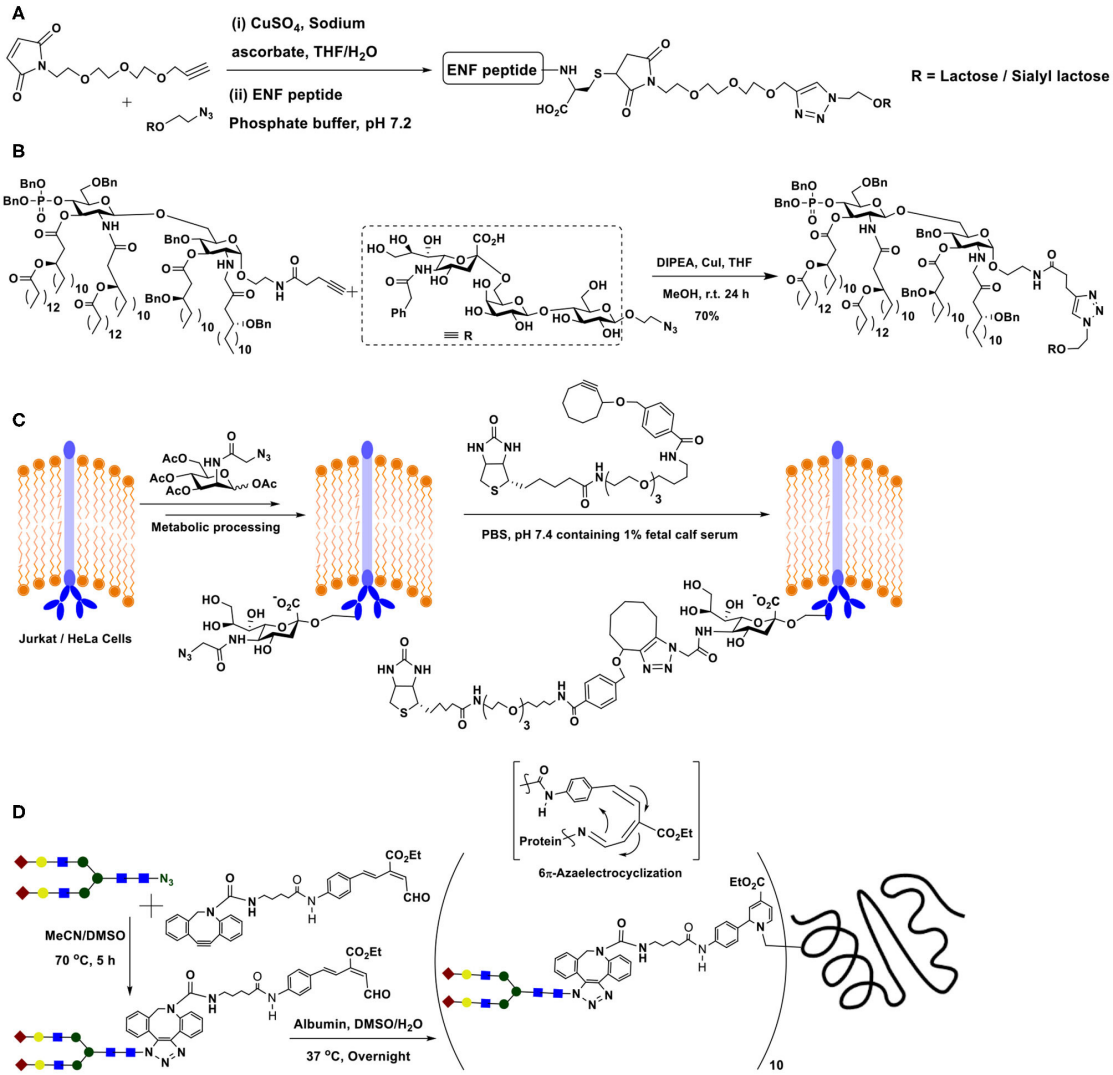
**(A)** Double conjugation involving Cu(I)-catalyzed azide-alkyne click reaction and thia-Michael reaction. **(B)** Sialyl-lactose derivative conjugation with modified lipid A through Cu(I)-catalyzed azide-alkyne click reaction. **(C)** Metal-free strain-promoted azide-alkyne cycloaddition reaction occurring at the metabolically engineered cell surface. **(D)** Double conjugation of metal-free azide-alkyne click and 6π-electrocyclization reactions, permitting the conjugation of biantennary complex *N*-glycan with human serum albumin.

The double conjugation method has been demonstrated in order to secure target glycopeptides in the case of anti-HIV peptide drug enfuvirtide, which is a 36 amino acid residue synthetic peptide. In order to improve the therapeutic potential of the drug, a thia-Michael reaction by maleimide-tethered glycosides was performed, for which the peptide was modified with cysteine residues at N- and C-terminals. Maleimide-tethered glycosides, in turn, possessed the triazole-linker linking the maleimide segment with glycoside segment intercepted further with ethylene glycol spacer (Scheme 12A). In this manner, chosen glycoside could be conjugated to both the termini of the peptide (Cheng et al., 2015).

Lipopolysaccharide glycolipids constitute as a major component of Gram-negative bacterial cell surface. The endotoxicity arising from a portion of the lipopolysaccharide, namely lipid A results from the phosphate moiety at the anomeric carbon, thus removal of this moiety enables loss of endotoxicity, even when retaining the immunostimulatory activity. The immunostimulatory activity prompts utilizing endotoxicity-free lipid A for conjugate vaccine preparation. The conjugation of tumor-associated carbohydrate antigen to modified lipid A was conducted, wherein the conjugation relied on azide-alkyne click reaction (Scheme 12B). Individual azide and alkyne tethered di- and trisaccharides were synthesized by chemical routes. The mono-phosphorylated glycolipid was tethered with alkyne, whereas a sialyl-lactose derivative, a tumor-associated GM3 antigen possessed azide moiety in the aglycon portion. The Cu(I)-mediated azide-alkyne click reaction occurred smoothly to afford the conjugate in good yields after column purifications (Tang et al., 2010).

Yet another important milestone in the azide-alkyne click reaction is the development of metal-free click reaction. The bio-orthogonal nature of the azide and alkyne components, the ability to undergo an irreversible reaction to form the triazole at room temperature and the biological compatibilities of these functional moieties warranted identification of the ability to conduct the reaction under a metal-free condition, the metal being incompatible under biological milieu. A solution for this was discovered by exploiting highly reactive internal alkynes, particularly cycloalkynes. The objective was achieved by identifying cyclooctyne as the alkyne component to react with azides. Deformation of bond angles about the alkyne moiety in cyclooctyne lead to a high ring strain-promoted reactivity. Further, the ability to tune the reactivity through electronic influences by substituents at proximal carbons added a greater advantage to exploit cyclooctyne as the most promising alkyne for metal-free click reaction with azides. Initial experiments were conducted on metabolic-incorporated sialic acid at the Jurkat cell surface, with the aid of appropriately modified mannosamine. Mannosamine was labeled with the azide moiety, which, in turn, permitted retaining the azide in the metabolically synthesized sialic acid at the cell surface. When cells incubated with cyclooctyne functionalized with a distal biotin, the azide-alkyne cycloaddition reaction occurred (Scheme 12C), as monitored comprehensively using fluorescein isothiocyanate-avidin mediated avidin-biotin interaction-enabled flow cytometry analyses (Agard et al., 2004).

Fine-tuning of the solubility and reactivity patterns warranted changes in the substituents exocyclic to cyclooctyne. Installation of *gem*-difluoro or *vic*-dimethoxy-substituent and replacing the aryl moiety with a nona-peptide, called FLAG peptide, were performed on the cyclooctyne and animal studies were conducted. Metabolic engineering of mice with mannosamine derivative led to the production of azide-labeled sialic acid at the cell surface, which was followed by treatment with the peptide-containing cyclooctyne. The click reaction occurred at the cell surface *in vivo*. The FLAG peptide present in the click component enabled flow cytometry analysis using an FITC-labeled anti-FLAG antibody.

Whereas, eukaryotic metabolic engineering of glycans and implementation of the click reaction required metal-free strain-promoted conditions, in the case of metabolic engineered bacterial glycans having azide and terminal alkyne functionalities at the bacterial cell surface underwent the click reaction under metal-promoted conditions. The originally identified metal-mediated click reaction occurs effectively under the cellular environment as well (Liang et al., 2017).

Further elegant developments on metal-free, strain-promoted glycoconjugation pertains to the reaction termed as “Riken click reaction.” The reaction is based on a 6π-azaelectrocyclization (Tanaka et al., 2011). Sustained work on the cyclooctyne derivative also showed that the presence of an endocyclic nitrogen atom in the cyclooctyne promotes the reaction with even higher reactivity. The method was utilized to conjugate a biantennary *N*-glycan on to albumin, which possesses multiple number of lysine sites. A terminal aldehyde functionalized *N*-glycan was prepared, through formation of 1,2,3-triazole by strain-promoted azide-alkyne click reaction. Aldehyde functionality was subjected to reaction with lysine residues of albumin, which subsequently underwent the 6π-electrocyclization to form conjugated enamine (Scheme 12D) (Sibgatullina et al., 2017).

### Thiol-Ene Reactions

**Scheme 13:**
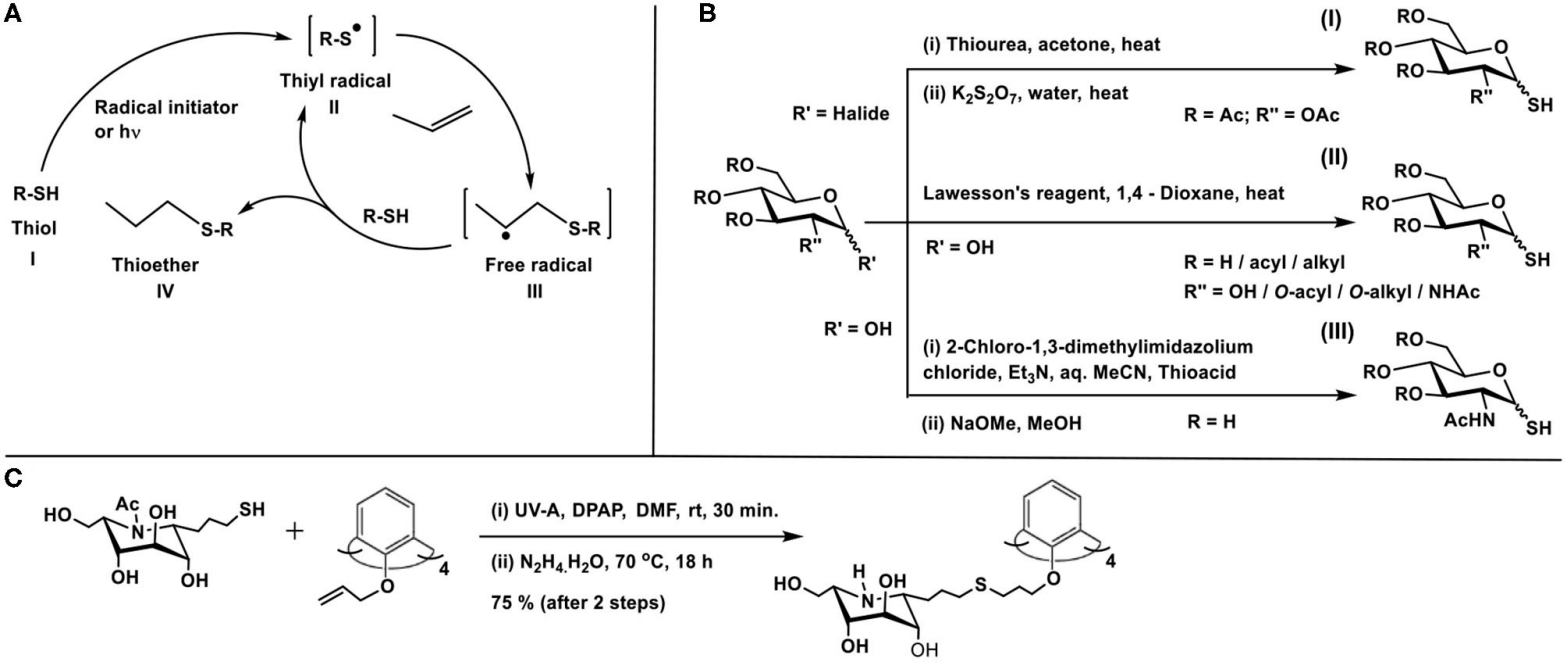
**(A)** Thiyl radical generation from thiol and reaction with ene to afford thioether product. **(B)** Methods of preparation of glycosyl thiols. **(C)** Glycoconjugation of calix-4-arene with thiol-tethered nojirimycin.

Thiol-ene conjugation is developed as a method in glycoconjugation chemistry prominently in the past decade. The reaction is characterized by the addition of a thiol across a double or triple bond, under activation by a radical initiator or by photochemical method. The mechanism of the reaction, described as early as in 1938 by Kharasch et al. proceeds through the generation of thiyl radical **II** upon activation of thiol **I**, by a peroxide (ascaridole) as the radical initiator in this work, the addition of which across the ene moiety occurs, leading to the formation of a new C-S bond, along with free radical at β-carbon to thioether functionality (**III**) (Scheme 13A). Abstraction of hydrogen from thiol **I** completes the reaction to afford thioether **IV** (Kharasch et al., 1938). Anti-Markovnikoff addition of the thiol moiety at less substituted carbon of the ene is a characteristic feature of this reaction. The reaction gained popularity in conjugation reactions immensely in the past decade (Hoyle et al., 2010), including glycoconjugations (Dondoni and Marra, 2012).

The presence of the thiol or the ene moiety exocyclic to the sugar ring is the most studied for these reactions. The procedure reported by Matta and co-workers is utilized prominently to synthesize the anomeric sugar hemithioacetals, namely sugar thiols (Matta et al., 1973). The preparation involves the sequence of (i) reaction of thiourea with a glycosyl halide and (ii) treatment of the same with potassium metabisulfite (Scheme 13BI). The method of Davis and co-workers is to utilize Lawesson's reagent by which sugar hemiacetal in 1,4-dioxane is converted directly to sugar thiol at elevated temperatures (Scheme 13BII). The method is developed for both protected and free hydroxy groups containing sugars, although the unprotected sugars and their thionation also lead to disulfide formation, which is then reduced to realize the sugar thiols (Bernardes et al., 2006). Further development to the sugar thiols pertains to the report on 2-acetamido sugars. Formation of sugar oxazoline, mediated by 2-chloro-1,3-dimethylimidazolium chloride, its reaction with thioacetic/thiobenzoic acid in aq. MeCN solution, followed by a base treatment affords sugar thiols in good yields (Scheme 13BIII) (Köhling et al., 2016).

On the other hand, the introduction of the ene-moiety is conducted through *O*-allylation at the anomeric carbon, a reaction which can be conducted on free sugars with ease. With the above tethering of sugars with reactive moieties, a number of reports utilized the thiol-ene reaction in glycoconjugation of chosen molecules and biomolecules. Following are the illustrative examples of thiol-ene-mediated glycoconjugations. The radical reaction proceeds in the presence of either a radical initiator, such as, azobisisobutyronitrile (AIBN) or under photocatalytic conditions, in the presence of an activator, such as, 2,2-dimethoxy-2-phenylacetophenone (DPAP). Irradiation with UV light (256 nm) or more benign visible light (365 nm) is utilized to initiate the reaction.

The thiol-ene reaction was developed as the key reaction to cluster imino sugars, that are known to be profound inhibitors of glycosidase and glycosyl transferase activities (Zelli et al., 2020). *O*-Allylation of phenolic moieties of the macrocyclic scaffold calix-4-arene (Groenen et al., 1991) affords the desired ene-moiety. On the other hand, judiciously chosen imino sugar is further tethered at *C*-1 carbon to secure *C*-iminoglycoside, namely, 1-allyl-1-deoxy-β-L-*ido*-nojirimycin. A thiol-ene reaction sequence on the nojirimycin derivative affords 1-(3-thiopropyl) nojirimycin derivative. Photochemical thiol-ene reaction of 1-(3-thiopropyl)nojirimycin with the *O*-allylated calix-4-arene, irradiated using a UV-A lamp (365 nm) and in the presence of DPAP as the activator, leads to the conjugation product calixarene L-ido-nojirimycin cluster (Scheme 13C).

**Figure 1:**
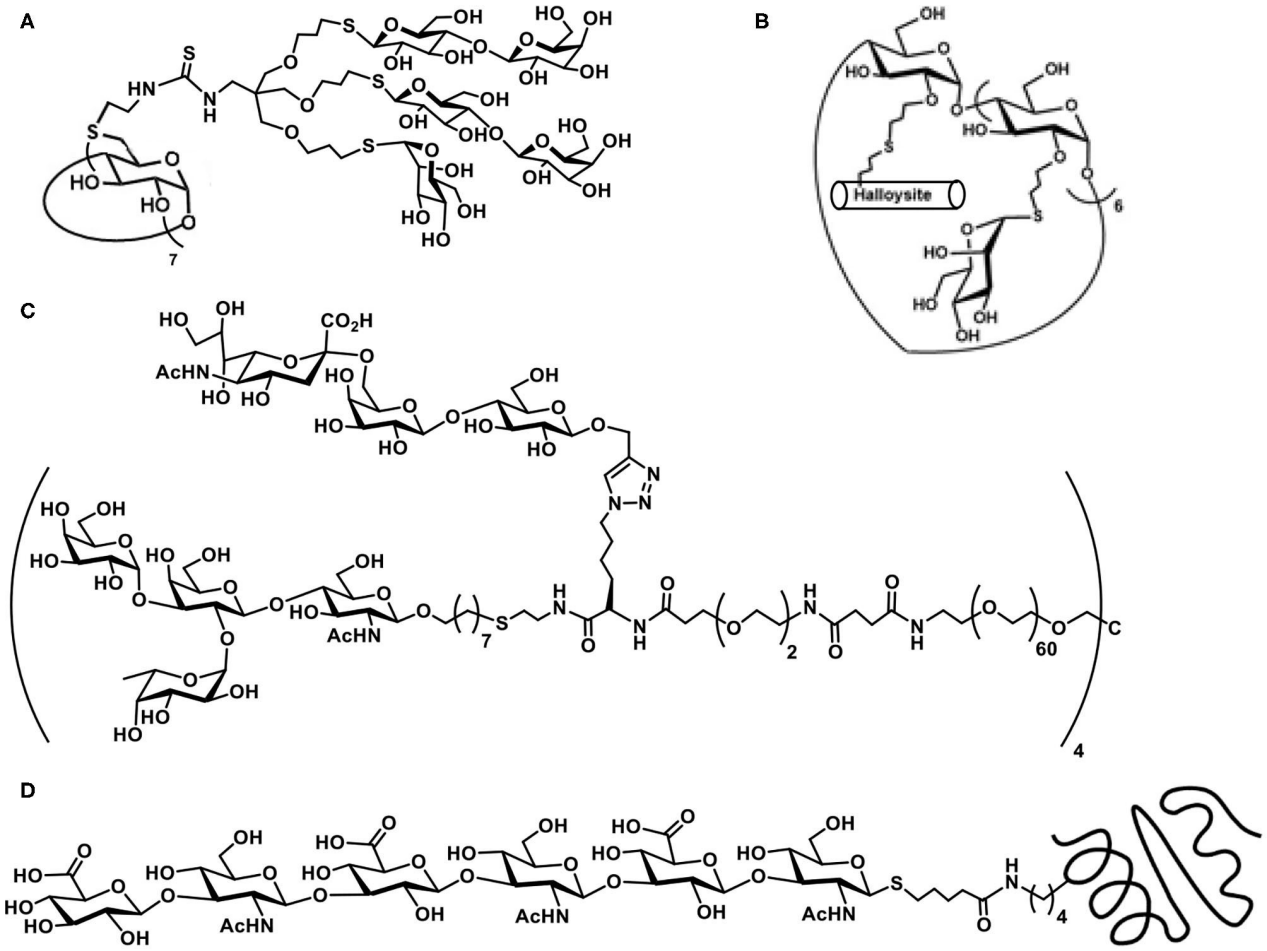
Molecular structures of: **(A)** β-cyclodextrin-cored multivalent heteroglycocluster; **(B)** dual drug-loadable mannopyranoside-halloysite-CD conjugate; **(C)** octavalent heteroglycocluster possessing two different types of glycoconjugation linkages; **(D)** hyaluronan hexasaccharide with thiol tether conjugated with engineered thermostable lipase TTL, through a thiol-ene reaction.

Exercising the efficient thiol-ene reaction on cyclodextrin molecular scaffolds has been demonstrated earlier (Fulton and Stoddart, 2000). The work of Benito et al. takes forward further to include sugar heterocluster synthesis, namely, the clusters possessing lactosyl and mannopyranosyl residues together in the same scaffold. Glycosyl thiols are attached to a tri-*O*-allylated pentaerythritol, in a sequential manner, under AIBN-catalyzed thiol-ene reaction to secure the hetero-tris-cluster. The tris-cluster is taken through further modifications with the aid of thiourea formation with per-(C-6)-cysteaminyl-β-CD to secure a multivalent heteroglycoconjugate (Figure 1A) (Gómez-García et al., 2012).

Functionalization of the CDs with sugar ligands is conducted beneficially by glycoconjugation. *O*-Allylation of the secondary hydroxy groups in β-CD, followed by conjugation with mannopyranosyl thiol was conducted by thiol-ene reaction under photochemical condition (Massaro et al., 2016). Out of available 14 secondary hydroxy groups, all but one underwent the thiol-ene reaction, whereas the remaining one allyl moiety was utilized to conjugate a thiol-tethered hollow tubular halloysite mineral (Figure 1B). Dual drug loading by halloysite and β-CD by two different drug molecules and their release properties were studied.

In a recent report, Cairo et al. demonstrate heteroglycocluster formation, wherein few arms of an octavalent glycocluster are presented with one of lactose/human blood group antigen tetrasacharide and the remaining arms of the glycocluster are installed with one of *N*-acetylneuraminic acid/3′/6′-sialyl lactose antigens. A combination of thiol-ene and azide-alkyne click reaction-based conjugations are adopted to realize the desired octavalent glycoconjugate (Figure 1C). The synthesis is achieved in a multi-step sequence, starting from basic building blocks, due to which diversity in the glycocluster size and variations in the ligand nature could be conducted easily. Validity of such designed heteroglycoclusters is demonstrated through their activity to enforce co-clustering of B-cell receptor and CD22 receptor, the functional consequence of which would be to induce the B-cell tolerance (Daskhan et al., 2018).

The thiol-ene reaction of an ene-tethered protein with a chosen hexasaccharide containing thiol moiety at the reducing end was demonstrated. The introduction of an *N*-pent-1-enoyl moiety at the ε-amino-group of lysine was achieved with the aid of stop-codon suppression method, whereas the hyaluronan hexasaccharide glycosyl thiol was secured through the method as given in Scheme 13B(III). The reaction of *N*-pentenoyl-tethered engineered thermostable lipase TTL from *Thermoanaerobacter thermohydrosulfuricus*, with the glycosyl thiol conducted under irradiation at pH 6 and a quantitative conjugation occurred (Figure 1D), as adjudged by HPLC-MS method (Köhling et al., 2016).

### Disulfide Bond-Based Glycoconjugations

**Scheme 14:**
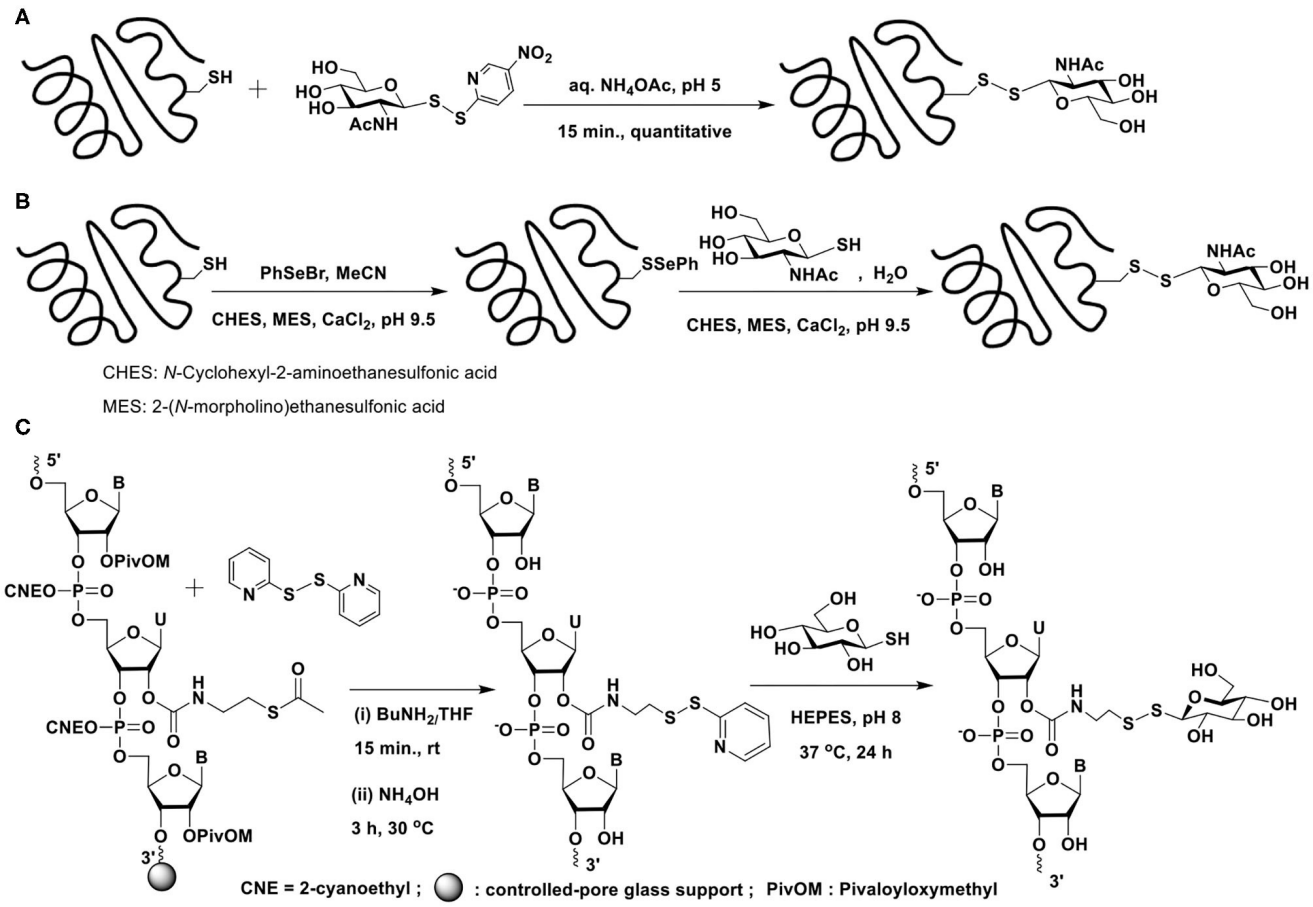
Glycoconjugation of: **(A)** BSA protein with an activated glycosyl moiety through mixed disulfide bond formation; **(B)** serine protease subtilisin *Bacillus lentus* mutant S156C (SBL-Cys156), modified with phenylselenylthio moiety, with a glycosyl thiol; **(C)** a 21-mer RNA sequence using glycosyl thiols.

Disulfide-based glycoconjugations have emerged as a resourceful methodology. In an early report, Boons and co-workers reported glycoconjugation on BSA protein, using disulfide bond formation (Macindoe et al., 1998). 2-Acetamido-2-deoxy-1-thio-β-D-glucose, prepared in 5 steps from D-glucose is treated with 2,2′-dithiobis(5-nitropyridine) (DTNP) in AcOH–water to secure the reactive disulfide bond possessing 5-nitropyridine-2-sulfenyl (pNpys) functionality. Reaction of this disulfide with cysteine in aq. NH_4_OAc (1 M) (pH 5) for 15 min. affords the mixed disulphide containing cysteine and 2-acetamido-2-deoxy sugar (Scheme 14A). Apart from extending the new mixed disulphide bond forming reaction on peptides, protein BSA, which contains a single cysteine, Cys-58, was subjected to glycoconjugation with the reactive disulfide reagent.

An alternate approach was devised by Davis et al. wherein the thiol moiety is activated as phenylselenenyl sulfide, which upon reaction with the thiol would afford the disulphide (Gamblin et al., 2004). Glycoconjugation of a protein would thus require the activation of cysteine thiol as selenenylsulfide (Scheme 14B). Reaction of the protein subtilisin *Bacillus lentus* (SBLS) having the activated thiol functionality with varied glycosyl thiols, prepared by using Lawesson's reagent afforded the glycoconjugated protein.

Apart from constitutionally differing monosaccharide glycosyl thiols, tri-, and heptasaccharide thiols were also conjugated to the protein employing the selenenylsulfide activation method. Further, the alternate strategy of activating the glycosyl thiol to the corresponding phenylselenyl sulfides and the reaction with thiol moiety of cysteine in proteins were conducted efficiently (Gamblin et al., 2004).

The disulfide bond formation strategy through activation of thiol with pyridyldisulfanyl substitution was utilized to glycoconjugate ribonucleotides (Scheme 14C) (Gauthier et al., 2019). The reaction of the 21-mer RNA having the activated disulfide tether with glucosyl / galactosyl thiols in HEPES buffer (pH 8) at 37°C for 24 h, afforded the corresponding glycoconjugated product in good yields.

### Suzuki-Miyaura Coupling-Based Glycoconjugations

Suzuki-Miyaura cross-coupling reaction pertains to the reaction of arylboronates with alkyl/aryl halides, mediated by a metal catalyst, leading to a C-C bond formation (Miyaura et al., 1979). One of the reactive moieties is halobenzene and introduction of this moiety onto the protein would facilitate coupling with the arylboronate component. The method advanced by Xie et al. aids in the introduction of 4-iodophenylalanine in proteins, with the use of an amber codon *Methanococcus jannaschii* ${\text{tRNA}}_{\text{CUA}}^{\text{Tyr}}$-tyrosyl-tRNA synthetase pair (Xie et al., 2004). Further development includes mutagenesis methods to express proteins containing unnatural amino acids in *Escherichia coli* (Young et al., 2010). Whereas, these methods require a molecular biology expertise, chemical approaches are also developed to install iodobenzene moiety into the amino acid residues. The reaction of *p*-bromomethyl iodobenzene with the thiol functionality in cysteine offers a direct method to incorporate the iodobenzene tether at the cysteine residues (Chalker et al., 2009).

**Scheme 15:**
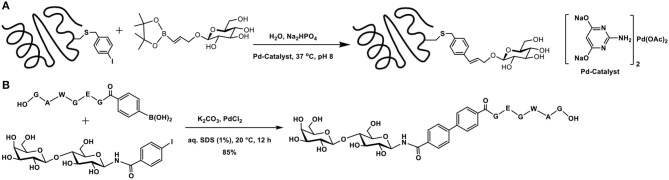
**(A)** Suzuki-Miyaura cross-coupling reaction of the functionalized SBL mutant protein with glucose-tethered vinylboronate. **(B)** Formation of biaryl core containing peptide and sugar segments.

The utility of bromo/iodoarene functionalized aromatic amino acids in Suzuki-Miyaura coupling with phenylboronic acids in aqueous buffer medium was demonstrated (Chalker et al., 2009). 2-Amino-4,6-dihydroxypyrimidine-Pd complex was used as the metal catalyst to mediate the reaction. This commercially available ligand mediates the cross-coupling reaction with proteins in a facile manner, in aqueous buffer solutions, at physiological temperature. Experiments with a number of bromo/iodoarene amino acids revealed that (i) chloro-substituent in place of bromide/iodide did not promote the reaction; (ii) *C*-terminal cysteine modified with *p*-iodobenzylthio functionality did not undergo the reaction, although internal cysteine in a peptide permits the cross-coupling reaction; and (iii) free-cysteine, as also glutathione, interfered, and the reaction did not take place. The applicability of the reaction was demonstrated through bioconjugation of mutant SBL-S156C protein from *Bacillus lentus*, which contains a single cysteine, the modification of which with *p*-bromomethyl iodobenzene afforded the required *p*-iodobenzyl functionality. Continuation of the work included coupling of the protein with glucose-tethered vinylboronate and the cross-coupled product was obtained in >95% conversion (Scheme 15A).

Glycoconjugation of a peptide fragment using Suzuki-Miyaura coupling was achieved (Lee et al., 2020). The efficiency of the reaction was investigated, with respect to the catalyst and base. Among bases, K_2_CO_3_ was found to be better, whereas among Pd-catalysts, PdCl_2_(dppf)_2_ was optimal. As a proof of concept of the method, the authors demonstrated glycoconjugation of a peptide with arylboronic acid and the sugar fragment containing aryl iodide, leading to the formation of the biaryl containing peptide and sugar segments (Scheme 15B).

Concerning glycoconjugations, Suzuki-Miyaura cross-coupling reactions occurring under aqueous milieu is sought after. Although not used widely for glycoconjugations, the method has seen initial success and wider applicability can be expected further.

### Metathesis Method of Glycoconjugations

A cross metathesis reaction facilitates the coupling of two individual alkene components to afford a cross-coupled product possessing a newly generated alkene, functionalized with the precursor individual alkene components. The reaction is mediated by metal catalysts, prominent among them are the generic Schrock catalyst, Grubbs, and modified catalysts. The reaction is adoptable in varying reaction media and conditions, added further with the compatibility of the reaction to many functional groups. The review by Aljarilla, López, and Plumet is illustrative on the metathesis methods valuable to secure carbohydrate containing derivatives and glycoconjugates (Aljarilla et al., 2010).

**Scheme 16:**
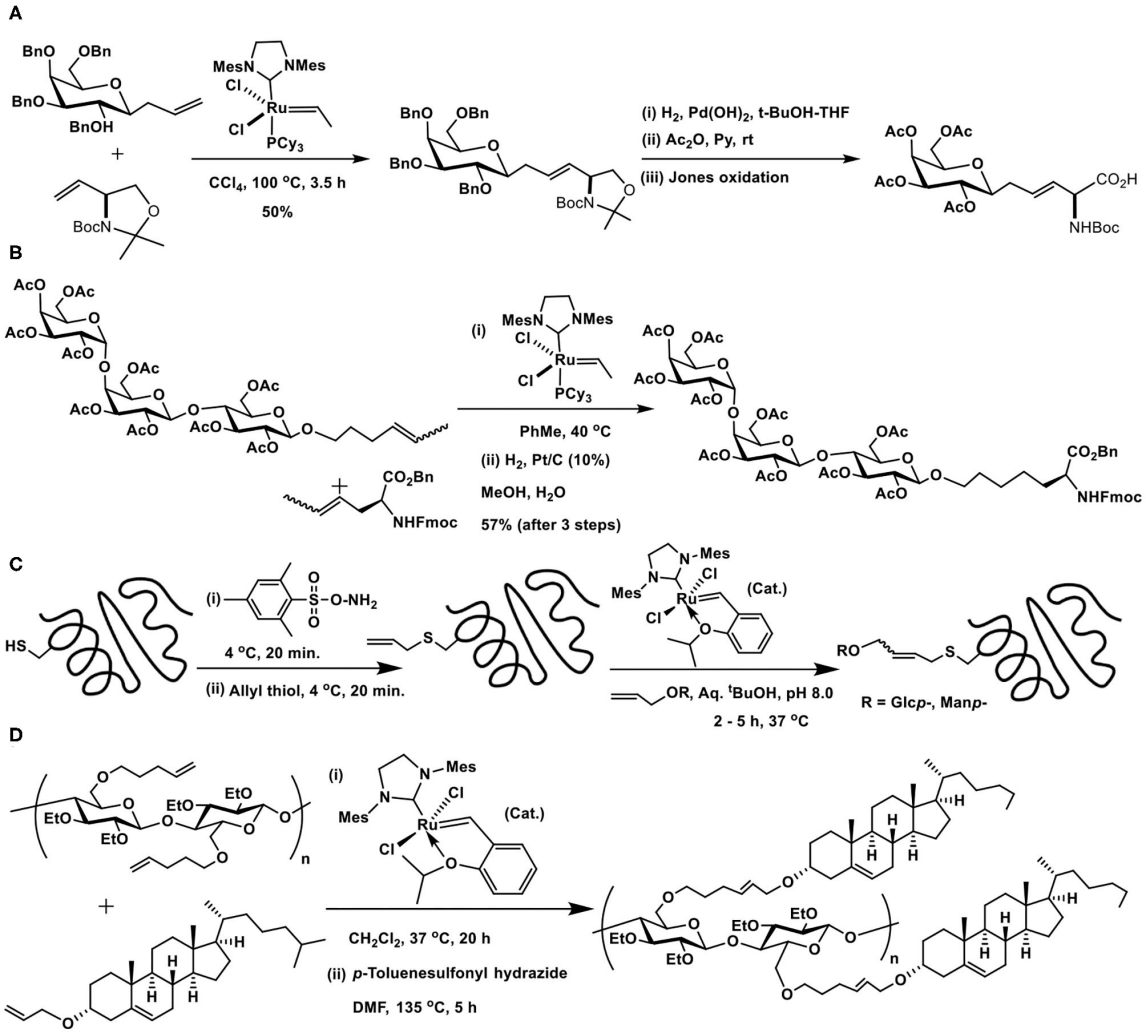
Cross metathesis reactions to the synthesis of: **(A)** glycosyl amino acid; **(B)** globotrioside (Gb3) conjugated amino acid; **(C)** glycosyl moieties incorporated SBL protein; **(D)** cholesterol moieties conjugated cellulose.

An early utility of the glycoconjugation pertains to the synthesis of *C*-glycosyl amino acid through reaction of allyl *C*-glucopyranoside with vinyl oxazolidine mediated by the second generation Grubbs catalyst, followed by further synthetic manipulations to afford *C*-glucopyranosyl amino acid (Scheme 16A) (Dondoni et al., 2001).

Multivalent sugar epitope conjugation to a peptide backbone was demonstrated by Wan et al. (Scheme 16B). The metathesis reaction was carried out from internal olefins, resulting from isomerization of the terminal ones in the presence of the catalyst, to afford the desired glycoconjugated amino acid. A few other glycosyl amino acids, including fucosyl GM1 heptasaccharide containing amino acid, were also accomplished (Wan et al., 2005).

*O*-Allyl trisaccharide, with → 3)-α-D-Rha*p*-(1 → 3)-α-D-Rha*p*-(1 → 4)-α-D-Gal*p*-(1 → sugar component, corresponding to the repeating unit of the *O*-polysaccharide of *B. cepacia*, a cystic fibrosis pathogen, was prepared and conjugated with protected L-allylglycine derivative using the cross metathesis conjugation method, as reported by Fauré et al. (2007).

Cysteine thiol in subtilisin *Bacillus lentus* (SBL) was modified with an allyl functionality and reacted with allyl glycosides (Lin et al., 2008). The required allylcysteine was secured by the reaction of cysteine with *O*-mesitylenesulfonylhydroxylamine to afford the dehydroalanine and a subsequent reaction with allylthiol. Second generation Hoveyda-Grubbs catalyst, devoid of the phophine ligand, was chosen as more compatible for bioconjugation with the protein. Aqueous ^t^BuOH was used to solubilize the catalyst and the reaction performed at <37°C at physiological temperature. Cross metathesis of *S*-allylcysteine-modified protein with gluco- and mannopyranosyl allyl glycosides afforded glycoconjugated protein in moderate yields (Scheme 16C).

The methodology was utilized to conjugate cholesterol to cellulose polymer, with the intent to solubilize otherwise water-insoluble drugs (Dong et al., 2019). The method of amorphous solid dispersion of drugs with the cholesterol-derivatized cellulose was adopted to retain a supersaturated amounts of the drug in the dispersion. In order to install the olefin functionality in the polymer, hydroxy groups at *C*-2 and *C*-3 carbons were masked with ethyl substituents, whereas the *C*-6 hydroxy group was derivatized by *O*-alkylation with 5-bromopent-1-ene. Hydroxy moiety in cholesterol was derivatized as *O*-acrylate, the reaction of the acrylate with alkene-tethered cellulose, mediated by second generation Grubbs-Hoveyada catalyst, afforded the conjugate in 84% yield. Subsequent partial hydrogenation using *p*-toluenesulfonyl hydrazide led the saturated linker connecting the cholesterol moiety with cellulose backbone (Scheme 16D).

### Vinyl Sulfone as Michael Acceptor in Glycoconjugations

**Scheme 17:**
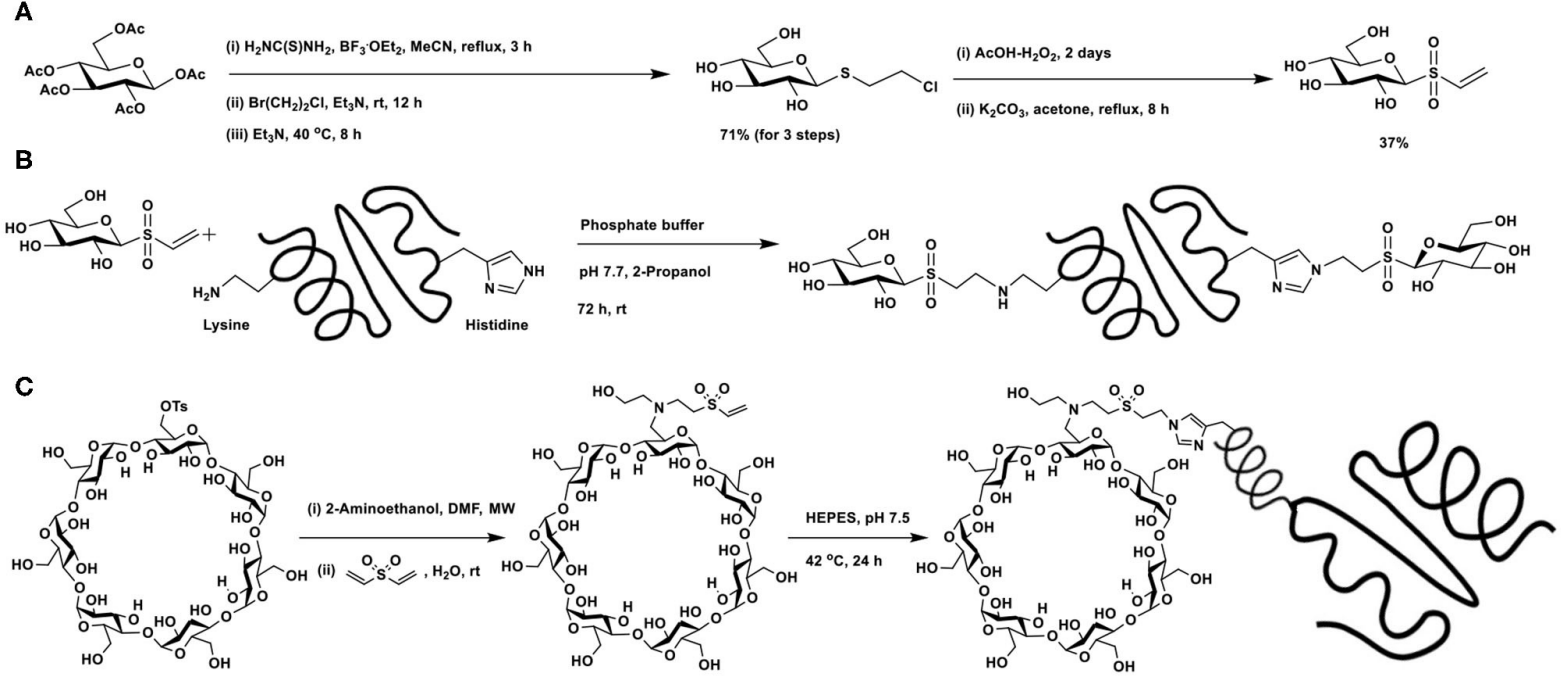
**(A)** Synthesis of glucosyl vinyl sulfone. Glycoconjugation of: **(B)** hen egg white lysozyme with glucosyl vinyl sulfone; **(C)** vinyl sulfone-functionalised β-cyclodextrin with nanobody cAb-An33.

The potential of vinyl sulfone as a Michael acceptor was demonstrated by Morales-Sanfrutos et al. (2010). Introduction of the vinyl sulfone functionality at the reducing end of a glycosyl moiety is shown in Scheme 17A. Resulting vinyl sulfone contains free hydroxy groups, making the synthon soluble in aqueous solutions. Protein hen egg white lysozyme, having 6 lysine and 1 histidine, none of free cysteine residues, was subjected to glycoconjugation with glycosyl vinyl sulfone in phosphate buffer (pH 7.7) (Scheme 17B). X-ray diffraction analysis, solved at 1.6 Å resolution, showed that in addition to lysine residues, histidine residue also underwent conjugation.

The vinyl sulfone-mediated glycoconjugation of cyclodextrin with recombinant antibody fragment raised against *Trypanosoma brucei* (del Castillo et al., 2014) was reported. The design requirement involved conjugation of the targeting device and drug encapsulant cyclodextrin, for which Michael addition reaction of vinyl sulfone with amine was undertaken (Scheme 17C). Mono-*O*-tosylation of primary hydroxy group, followed by reaction with 2-aminoethanol afforded *N*-alkylation intermediate, the reaction of which with divinylsulfone led to the formation of vinylsulfone-functionalized CD. The mild reaction conditions, aqueous stability and lack of by-product formation are featuring favoring Michael additions with vinylsulfones. A poly-His-tagged nanobody, namely, cAb-An33 was subjected to conjugation through Michael addition with vinylsulfone moiety and gave the overall labeling of 47%. Stronger nucleophilic character of His-residue at the polyHis-tag was rationalized as the mode of His-conjugation. Inclusion of the drug nitrofurazone at the cyclodextrin cavity, followed by functional biological assay on *T*. *brucei* culture showed that the drug conjugate inhibited the parasite growth, whereas the drug-nanobody alone did not inhibit the growth.

### Vinylsulfoxide Method of Glycoconjugation

Vinyl sulfoxide functionality adds another feature beneficial to the reactivity. The basis for the selectivity was that the approach of the nucleophile would occur from the opposite side of the lone pair of electrons on sulfur in the ground state. Michael additions on sugar vinyl sulfoxides might thus be anticipated to occur not only easily, but also with high stereoselectivities.

**Scheme 18:**
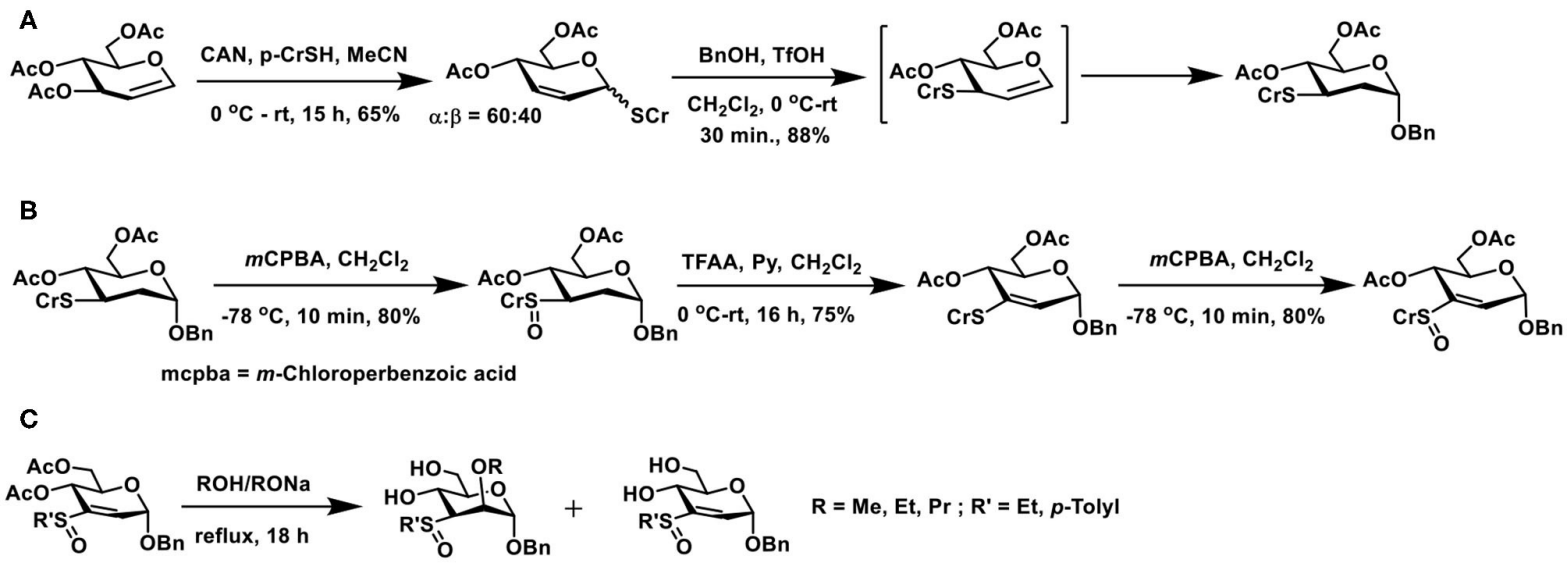
**(A)** Ferrier reaction of 3,4,6-tri-*O*-acetyl glycal to afford 2,3-unsaturated thioglycoside and a subsequent conversion of thioglycoside to the *O*-glycoside. **(B)** Formation of sugar vinyl sulfoxide through Pummerer rearrangement and an oxidation. **(C)** Reaction of oxygen nucleophiles with sugar vinyl sulfoxide.

Michael addition of sugar vinyl sulfoxides provides a newer route to glycoconjugations. The method to prepare a defined sugar vinyl sulfoxide turned out to be first of the effort to realize this synthon. Unsaturated sugars possess rich reactivities, an elegant example is the Ferrier reaction. The reaction relies on Lewis acid activation of 1,2-unsaturated sugars having ester protecting groups and, in the presence of a nucleophile, the reaction leads to the formation of 2,3-unsaturated sugars with concomitant addition of the nucleophile at the anomeric carbon (Ferrier and Prasad, 1969). When the reaction is conducted in the presence of thiol nucleophile, a 2,3-unsaturated thioglycoside results. The reaction of 1,2-unsaturated sugar with thiol in the presence of catalytic ceric ammonium nitrate (CAN) affords 2,3-unsaturated thioglycoside as the major product (Scheme 18A). The addition of the nucleophile across the double bond also occurs so as to afford a 2-deoxy thioglycoside.

2,3-Unsaturated thiogycoside can, in turn, be activated by another acid-catalyzed migration of *C*-1 thioether moiety to *C*-3 and a concomitant glycosylation to afford 2-deoxy glycoside. Catalysis by Brønsted acid is feasible, in the absence of which, the 1 → 3 shift does not occur, indicating the activation of the olefin by acid induces the migration of anomeric thioether moiety to *C*-3 carbon. A double bond migration under the acidic condition and addition of alcohol leads to the product, namely 2,3-dideoxy-3-thiotolyl glycopyranoside, as the α-anomer (Mukherjee and Jayaraman, 2011). Formation of this product was sufficient in order to transform to the required vinyl sulfoxide synthon, through implementation of known synthesis.

Controlled oxidation of 3-deoxy-3-thioether derivative with *m*-chlorobenzoic acid afforded sulfoxide (Scheme 18B). Sulfoxides are excellent synthons for Pummerer rearrangement. The rearrangement occurs by activation of sulfoxide by an acid anhydride, in the presence of a base, leading to either a thioacetal or, under conditions, a vinyl thioether. Rearrangement of 3-deoxy-3-sulfoxide synthon, when treated with trifluoroacetic anhydride (TFAA)/pyridine at room temperature overnight, afforded vinyl sulfide, in a good yield. The vinyl sulfide, in turn, was subjected to an oxidation, to afford the vinyl sulfoxide. The newly-formed sugar vinyl sulfoxide was a diastereomeric mixture at sulfoxide.

Nucleophilic additions on sugar vinyl sulfoxides can be implemented using oxygen, nitrogen, carbon, and sulfur nucleophiles. Using oxygen nucleophiles, the conjugate addition occurred to form the corresponding adduct (Scheme 18C) under reflux conditions. The addition occurred axial at *C*-2 carbon, so as to form the *manno*-configured product. The ester protecting groups deprotected under the reaction condition to form the corresponding alkoxides. Alkoxides appeared to hinder the subsequent addition reaction, leading to the isolation of the starting vinylsulfoxide synthon. Ethyl and cresyl (*p*-tolyl) sulfoxides underwent addition reactions to a similar extent (Mukherjee and Jayaraman, 2012).

**Scheme 19:**
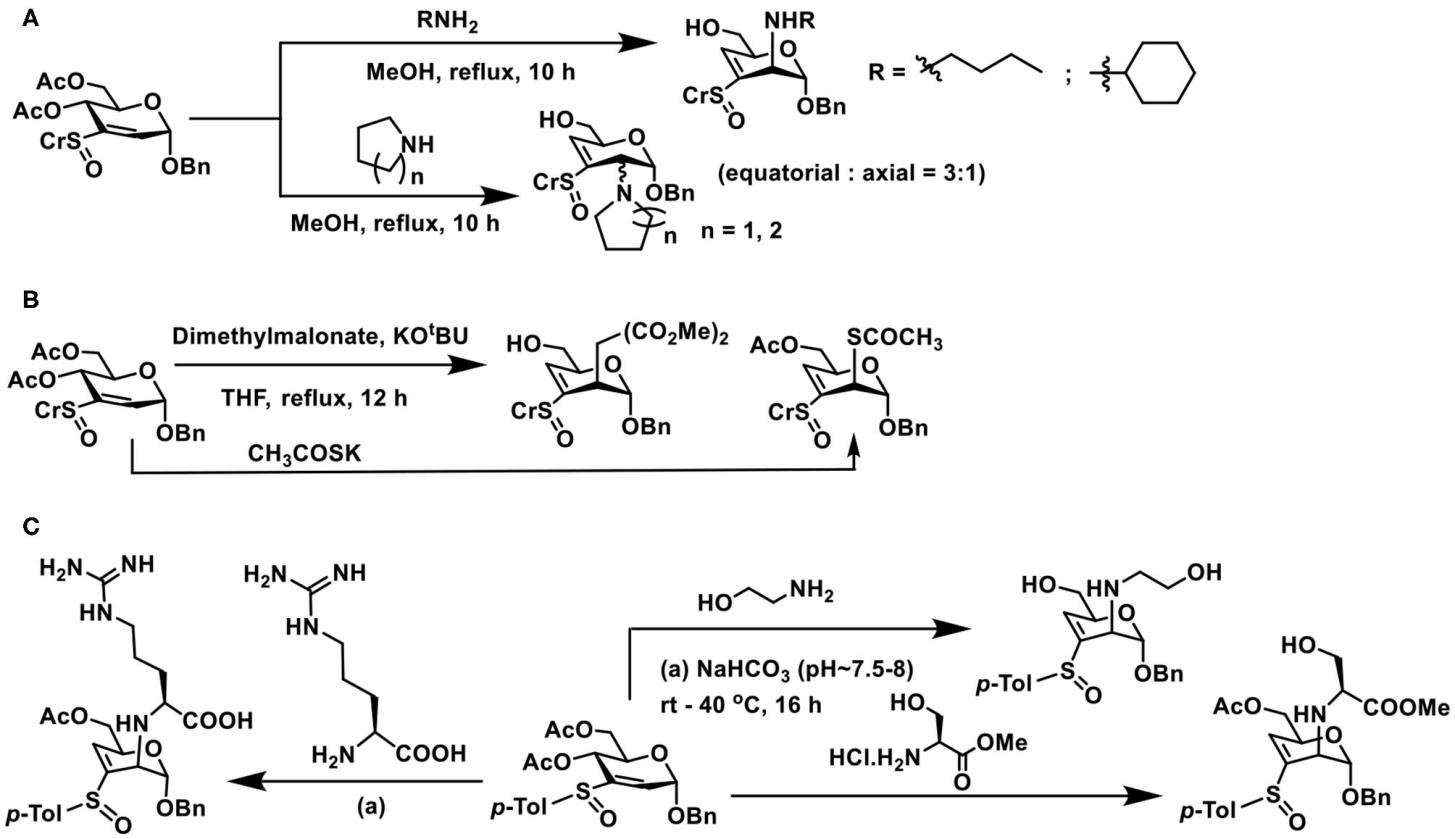
Reactions of: **(A)** amines on sugar vinyl sulfoxides; **(B)** sugar vinyl sulfoxide with carbon and sulfur nucleophiles; **(C)** vinyl sulfoxide with 2-aminoethanol, serine and arginine.

Nucleophilic additions of nitrogen nucleophile to sugar vinyl sulfoxides were different from that of the oxygen nucleophiles. Whereas, the addition occurs at the *C*-2 carbon of vinyl sulfoxide, it also leads to an elimination reaction and affords *C*-3—*C*-4 unsaturated vinyl sulfoxide, in an S_N_2′- type addition-elimination reaction. Initial reactions of sugar vinyl sulfoxide with primary amines, such as, *n*-butyl amine or cyclohexyl amine, showed that the addition occurred at the axial face to form the *manno*-configured product, possessing a double bond at *C*-3–*C*-4 carbons (Scheme 19A). An external base, such as, NaHCO_3_, was not required with amine nucleophiles for the addition reaction. Yet the reaction required a higher temperature for the conversion, which otherwise required few days for the product formation at room temperature. Piperidine, pyrrolidine, and morpholine as nucleophiles provided addition-elimination products, wherein the nucleophile added at both axial and equatorial faces, the major product being the equatorial orientation (Mukherjee and Jayaraman, 2013).

Reaction of sugar vinyl sulfoxide with carbon nucleophile and sulfur nucleophile followed a similar reaction outcome as amines. Dimethyl malonate in THF, the presence of KO^t^Bu, afforded the addition-elimination product in 94% yield. Whereas, potassium thioacetate in THF, used as sulfur nucleophile, without an external base afforded the addition product in 96% yield after 12 h (Scheme 19B). Acetate moiety at *C*-6 either retained or deprotected under the conditions used.

The above observations reiterate that the addition-elimination reaction is the preferred mode with nitrogen, sulfur, and carbon nucleophiles. The reaction requires a basic condition and addition leads to *manno*-configuration largely, although with bulky secondary amines, stereoselectivity was poor. Further, no other reagent was required in the conversion of the vinyl sulfoxide to the conjugate addition product. Protic solvents and aqueous solutions are also feasible, thereby making the reaction compatible in a range of organic and aqueous solutions.

Conjugate addition with amine appeared appealing among the nucleophiles used in the reaction, thereby opening a rich avenue for glycoconjugations on to peptides and proteins. The ε-amine moiety in lysine, along with α-amine in amino acids, provides an easy access to conjugate sugar vinyl sulfoxides. Early reaction of 2-aminoethanol as the Michael donor, addition occurred in a methanolic solution, wherein 2,3-unsaturated vinyl sulfoxide was converted to 3,4-unsaturated vinyl sulfoxide with nucleophile addition at *C*-2 in the *manno*-configuration. The reactivity with amine also retained in the presence of a base (NaHCO_3_), though hydrolysis of acetate at *C*-6 carbon occurred (Scheme 19C). Concerning free amino acids, reactions with serine and arginine, in aqueous methanolic solution (MeOH/Water 1:1), under base-promoted conditions, led to the conjugation products as only products, with *manno*-configuration at *C*-2. With both these amino acids, only the α-amine moiety participates in the reactions, hydrolysis of acetate occurred under the conditions (Sarkar et al., 2019).

**Scheme 20:**
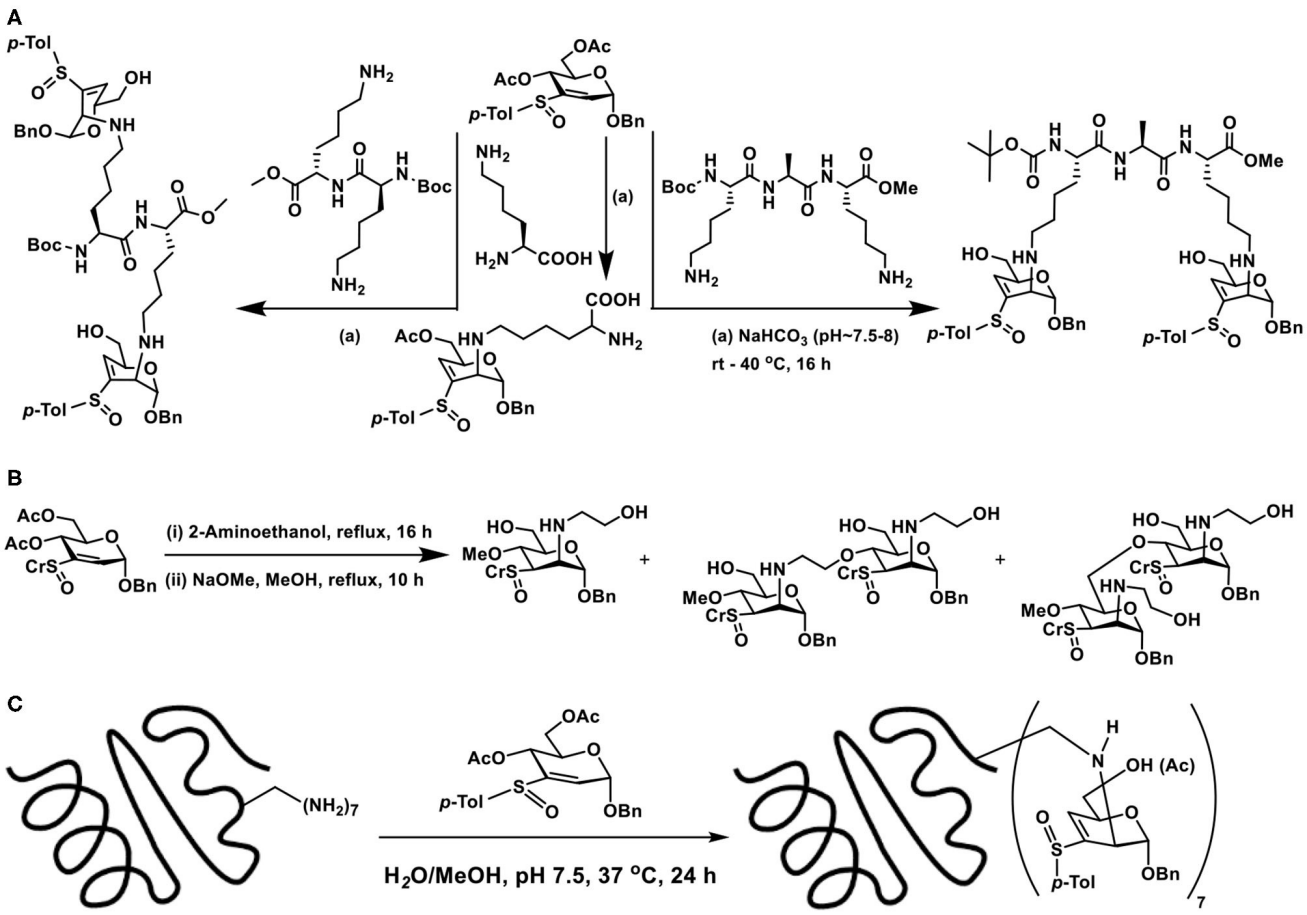
**(A)** Reaction of vinyl sulfoxide with lysine, di- and tripeptides. **(B)** Conjugation of vinyl sulfoxide with amine initially, followed by the alkoxide nucleophile. **(C)** Glycoconjugation of lysozyme with sugar vinyl sulfoxide.

Lysine, Lys-Lys, and Lys-Ala-Lys, di-, and tripeptides, respectively, were subjected to reaction with vinyl sulfoxide, following a similar protocol, namely, an aqueous methanolic solution containing the reactants and base was stirred for 16 h at 37°C, followed by purification (SiO_2_). Conjugate addition products formed as the only product of the reaction (Scheme 20A). Other than that, unreacted vinyl sulfoxide remained with free hydroxy groups formed after hydrolysis. Formation of glycopeptide conjugation products under benign conditions reiterated sugar vinyl sulfoxide as a new synthon in glycoconjugations.

Formation of yet another vinyl sulfoxide upon conjugation prompted possibility to conduct another conjugate addition. When large excess of amine was used in the reactions, conjugation of amine did not occur. Interestingly, when NaOMe/MeOH was used, conjugate addition by methoxide occurred. The basic condition also promoted deprotonation of available alcohol moieties and these alkoxides also underwent the conjugate addition reaction, leading to a mixture of products (Scheme 20B). Though the second conjugation addition was not optimized, the observations illustrate that *C*-3–*C*-4-unsaturated vinyl sulfoxide undergoes the reaction and opens up the possibility to oxygen nucleophiles to an extent.

The applicability of the method was extended to proteins possessing multiple lysine sites. For this purpose, readily available egg white lysozyme was chosen. Lysozyme acts on peptidoglycan layer bacterial cell wall components of gram-positive and gram-negative bacteria and hydrolyses glycans at *N*-acetylmuramic acid and *N*-acetyl glucosamine (Qasba and Kumar, 1997). It is an enzyme present in saliva, milk and tears and possesses antimicrobial properties (Jolles and Jolles, 1984). Constituted with 129 amino acid residues, it contains 6 lysine residues at 1, 13, 33, 96, 97, and 116 positions and no cysteines, though cystine is present. Glycoconjugation, with excess molar equivalents of vinyl sulfoxide *per* lysine residue, was carried out in aq. methanolic solution, at pH 7.5, maintained using NaHCO_3_. The reaction was conducted at 37°C for 24 h, reaction mixture lyophilized, washed with methanol few times, in order to remove excess sugar vinyl sulfoxide, resulting protein was re-suspended in aq. solution and subjected to a series of analysis (Scheme 20C). MALDI-TOF mass spectrometry of the reaction product showed intense molecular ion peak at *m/z* 17,016.0, an increased molecular mass compared to native lysozyme, with molecular mass of *m/z* 14,412.2. This increase in molecular mass corresponded to addition of 7 sugar vinyl sulfoxide moieties on to the protein. In conjunction with studies on peptides, the ε-amino moiety of lysine was estimated to undergo the glycoconjugation. The addition-elimination pattern of the reaction occurred, so as to present the sugar component as vinyl sulfoxide, with *C*-3–*C*-4 unsaturation. The acetate moiety at *C*-6 carbon was either present or hydrolysed to free hydroxy moiety. Peaks at *m/z* 17,057, 17,015, 16,973, 16,931 in LC-MS corresponded to seven modifications in lysozyme, followed by hydrolysis of the acetate moieties. In addition, a broadly distributed peaks at *m/z* 34,141.8 was also observed, corresponding to the dimer of the modified lysozyme, as in the case of the native lysozyme (Onuma and Inaka, 2008).

Identification of sites of modification warranted a trypsin digestion analysis. Trypsin is a protease enzyme which hydrolyses the peptide bond at positively charged amino acids, namely, lysine and arginine, at the C-terminal. Trypsin digestion can be conducted by an in-gel route, by which minced gel pieces are subjected to dehydration, re-hydration in reduction solution, alkylation solution and yet another dehydration, followed by treatment with trypsin. When the trypsin digestion analysis was conducted on native lysozyme, the protease activity led to peptide bond hydrolysis at lysine and arginine sites. Similar analysis on glycoconjugated lysozyme revealed a different course of hydrolysis, wherein only the peptide bonds at arginine sites cleaved and none of the effect on peptide bonds at the lysine sites. Peptide bond cleavage occurred at eleven arginine present in the protein. The role of glycoconjugation on the lysine sites of the protein affected the peptidase activity. Inaccessibility of the binding site by the enzyme as a result of the conjugation might affect the peptidase activity. Whereas, the available arginine sites in lysozyme undergo the enzymatic cleavage, as these sites remained without a conjugation.

Glycoconjugation of lysozyme also affects the functions of the protein. Lysozyme causes lysis of bacterial cell wall components, thereby providing antibacterial protection to the host. The bactericidal activity of lysozyme coupled with its thermal stability finds immense use in many utilitarian applications (Qasba and Kumar, 1997). The sugar vinyl sulfoxide conjugated lysozyme was subjected to antimicrobial assays on gram-negative *E.coli* bacterial cells and compared with that of the native lysozyme. At a concentration of 60 μg mL^−1^ and monitoring for up to 6 h, the native and the modified lysozymes showed a significant and comparable lysis of the bacterial cells. These preliminary observations suggested that glycoconjugations did not affect the antimicrobial property of lysozyme and might offer a promise for applications of glycoconjugated protein (Sarkar et al., 2019).

The above discussion illustrates that sugar vinyl sulfoxides are emerging synthons for glycoconjugations. Whereas, oxygen nucleophile affords conjugate addition product, that in the case of carbon, sulfur and nitrogen nucleophiles, an addition-elimination occurs and the product presents yet another vinyl sulfoxide. Sugar vinylsulfoxide-based glycoconjugation opens up a newer avenue to conjugate sugars on to peptides and proteins.

## Conclusion

The above narration of the chemical methods points to the fact that glycoconjugation of chosen sugars on to proteins and other biomolecules has emerged as an important area of research. Unexpected immune response to the linker connecting the sugar epitope with the carrier protein can be a serious impediment, as illustrated with LPS-based vaccine development against wild-type meningococcal bacterial strain (St. Michael et al., 2014). Whereas the conjugation methods are well-developed, site selectivity issues are highly demanding, and many methods lack the ability to conjugate the sugars at a pre-determined site, for example, in a protein with several lysine sites. Native chemical ligation partly provides a solution, utilizing the cysteine as the chemical handle to install a glycosylated amino acid at a defined location. The technique was further demonstrated for *C*-terminal ligation of chosen proteins with glycosides, mediated by intein-generated thioester, presenting a cysteine residue at the *N*-terminal of intein (Tolbert and Wong, 2000). Innovative methods to install glycosylated amino acid in a growing protein during biosynthesis was elegantly developed by Hecht (Fahmi et al., 2007). Importantly, sustained works have established a glycoprotein synthesis as an integral part of the translational machinery, as opposed to post-translational modification of proteins with glycoside components. This approach paves a way for the complete control of the site-selectivity, not easily achievable by other methods. Whereas, such biochemical works have evolved, chemical methods that offer a high control over the site of glycoconjugation *a priori* are not available to any reasonable extent. Secondly, chemical methods that do not require prior modifications of the macromolecular scaffolds are desirable, as are the conjugations occurring under reagentless conditions. These and more challenges should be driving the future developments in the glycoconjugations research.

## Author Contributions

NJ conceived the article. BS and NJ compiled and wrote the article. Both authors contributed to the article and approved the submitted version.

## Conflict of Interest

The authors declare that the research was conducted in the absence of any commercial or financial relationships that could be construed as a potential conflict of interest.
